# Definition of Human Apolipoprotein A-I Epitopes Recognized by Autoantibodies Present in Patients with Cardiovascular Diseases*[Fn FN2]

**DOI:** 10.1074/jbc.M114.589002

**Published:** 2014-08-28

**Authors:** Priscila Camillo Teixeira, Axel Ducret, Philippe Ferber, Hubert Gaertner, Oliver Hartley, Sabrina Pagano, Michelle Butterfield, Hanno Langen, Nicolas Vuilleumier, Paul Cutler

**Affiliations:** From the ‡ Pharma Research and Early Development, Roche Innovation Center, 4070 Basel,; the § Department of Genetics and Laboratory Medicine, Division of Laboratory Medicine, 1205 Geneva University Hospitals, 1205 Geneva, and; the ¶ Department of Immunopathology, Faculty of Medicine, University of Geneva, 1205 Geneva, Switzerland

**Keywords:** Autoimmune Disease, Cardiovascular Disease, Epitope Mapping, Mass Spectrometry (MS), Protein Purification, Anti-apolipoprotein A-I, Immunoreactive Peptides

## Abstract

Autoantibodies to apolipoprotein A-I (anti-apoA-I IgG) have been shown to be both markers and mediators of cardiovascular disease, promoting atherogenesis and unstable atherosclerotic plaque. Previous studies have shown that high levels of anti-apoA-I IgGs are independently associated with major adverse cardiovascular events in patients with myocardial infarction. Autoantibody responses to apoA-I can be polyclonal and it is likely that more than one epitope may exist. To identify the specific immunoreactive peptides in apoA-I, we have developed a set of methodologies and procedures to isolate, purify, and identify novel apoA-I endogenous epitopes. First, we generated high purity apoA-I from human plasma, using thiophilic interaction chromatography followed by enzymatic digestion specifically at lysine or arginine residues. Immunoreactivity to the different peptides generated was tested by ELISA using serum obtained from patients with acute myocardial infarction and high titers of autoantibodies to native apoA-I. The immunoreactive peptides were further sequenced by mass spectrometry. Our approach successfully identified two novel immunoreactive peptides, recognized by autoantibodies from patients suffering from myocardial infarction, who contain a high titer of anti-apoA-I IgG. The discovery of these epitopes may open innovative prognostic and therapeutic opportunities potentially suitable to improve current cardiovascular risk stratification.

## Teaser

**Background:** Autoantibodies to apoA-I can be considered markers and mediators of cardiovascular disease.

**Results:** Methodologies for identification of human apoA-I epitopes are described. Two novel immunoreactive peptides were recognized by autoantibodies from cardiac patients.

**Conclusion:** We developed a new procedure to isolate, purify, and identify apoA-I epitopes.

**Significance:** Developing new epitope mapping approaches opens new avenues for biomarkers and therapy.

## Introduction

In the field of cardiovascular research, not only are new therapeutic approaches required, but also better risk stratification tools. Current clinically based risk stratification methods, such as Framingham, Procam, and SCORE have been shown to be insufficient in predicting caradiovasculare disease (CVD).^3^ For instance, 60% of events happen in patients identified as being at low or moderate risk (1) and 30–50% of patients at risk are identified by a major event (2). Discovering new risk factors may also represent an opportunity to identify new pathways associated with CVD and clinical targets. Autoimmunity has started to be recognized as a cardiovascular risk factor, because several autoimmune conditions and immunological disorders, such as rheumatoid arthritis, systemic lupus erythematosus, and antiphospholipid syndrome, are characterized by enhanced atherosclerosis and consequently higher cardiovascular morbidity and mortality rates (3–10). There is also accumulating evidence showing that humoral autoimmunity might play an important role in CVD, by modulating atherogenesis. Several autoantigens and their respective autoantibodies have been considered to be associated with CVD, such as anti-HSP-60, antioxidixed LDL, anti-βGPI, and anti-HDL/apolipoprotein A-I IgG (11–15). More precisely, autoantibodies to apolipoprotein A-I (anti-apoA-I IgG), the major protein fraction of high density lipoprotein (HDL), have been shown to be both a marker and a mediator of cardiovascular disease, promoting atherogenesis and unstable atherosclerotic plaque through the engagement of innate immunity receptors (12, 16–18). Recent studies demonstrate that anti-apoA-I IgGs are raised in many diseases associated with a high cardiovascular risk, such as systemic lupus erythematosus, acute coronary syndrome, rheumatoid arthritis, severe carotid stenosis, and end-stage renal disease (19–28). Because autoantibody responses can be polyclonal and targeting multiple epitopes, it is possible that some of the clinical observations may result from the activity of different autoantibodies to different apoA-I epitopes. Thus, the precise identification and characterization of specific epitope(s) recognized by these autoantibodies is essential to gain a better understanding of their pathological role and potential use as biomarkers of cardiovascular risk prediction. Such information may also clarify the reasons for the presence of autoantibodies against a high abundant plasma protein, such as apoA-I, potentially highlighting novel targets for therapeutic intervention.

ApoA-I is synthesized in the liver and intestine, the mature form being found in circulation as a 28-kDa (243 amino acid) single polypeptide, lacking both glycosylation and disulfide linkages (29). Aside from the N-terminal 44 amino acids, the sequence appears to be organized into α-helical segments of 22 amino acids and two 11-mer repeats separated by proline residues (30). These helices are predicted to be amphipathic, with a hydrophobic face that mediates lipid interactions and a polar face that interacts with water (31, 32). Different established techniques have been adapted for the separation and/or quantitation of apolipoproteins (33–38). Purification of apolipoproteins is, however, complicated due to their amphipathic properties and the presence of a number of different isoforms (39). We have applied a conventional approach, using ultracentrifugation and gel filtration techniques, to isolated human apoA-I from healthy blood donors (40), followed by thiophilic interaction chromatography (TIC) (41), creating a high purity apoA-I suitable for subsequent epitope determination. A similar procedure, using sulfhydryl covalent chromatography, was previously used to isolate particles containing apoA-I (no cysteines in the sequence) from apoA-II in human plasma (42, 43).

ApoA-I epitope(s) could include linear, continuous and discontinuous conformational epitopes, macromolecular complex epitopes, neo-epitopes, and mimotopes. Moreover, autoepitopes can be hidden in the structure of the native antigen, exposed on the surface of apoptotic cells, or represented by structures mimicking the epitope (44). We established an approach for epitope definition based on limited proteolysis of the purified apoA-I, generating peptide maps that could be resolved by chromatography and subsequent identified by mass spectrometry. An advantage of such an approach is that the peptides could be transferred to solid phases to test the autoantibody immunoreactivity. Depending on the endoproteases used, the fragments generated can have different sizes. Usually proteolysis by LysC or ArgC generates larger fragments compared with trypsin or chymotrypsin, increasing the probability to obtain antigenic peptides. We rationalized that larger fragments generated by the enzymatic digestion at lysine or arginine would increase the probability of maintaining secondary structure integrity, and also antigenicity. Therefore, we applied in this study a set of methodologies and procedures to isolate, purify, and identify endogenous epitopes present in the apoA-I.

## EXPERIMENTAL PROCEDURES

### 

#### 

##### Patient Sera Samples

The patient samples used in this study were derived from a previously published prospective single-center study of 138 clinically well characterized patients presenting acute chest pain at the emergency room (25). The study was approved by the local ethics committee and performed according to the Declaration of Helsinki (18). Sera from 140 healthy donors were used to assess a reference range of anti-apoA-I IgG values (described below). The upper reference range was set at the 97.5th percentile of the distribution curve, *i.e. A*_405 nm_ = 0.64. Values ranged from 0.15 to 0.71. A serum sample was therefore considered positive if the absorbance value was above 0.64. We selected three different samples classified as high titer for anti-apoA-I IgG (positive control samples) and three different samples classified as low titer for anti-apoA-I IgG (negative control samples) to perform the experiments. All patient samples were collected after percutaneous coronary intervention within the first 24 h after hospitalization. After collection, serum samples were frozen in aliquots at −80 °C until required for analyses.

##### Determination of Human Antibodies to ApoA-I by ELISA

Anti-apoA-I IgG autoantibodies in plasma/serum (here referred to as the “samples”) were measured as described previously (19, 45), with minor modifications. Maxisorb plates (Nunc) were coated with purified and delipidated human apoA-I, diluted in carbonate buffer, pH 9.7 (20 μg/ml; 50 μl/well), for 1 h at 37 °C. The same procedure was used for peptide and enzymatic fragments. After three washes with PBS, 2% BSA (w/v) (100 μl/well), all wells were blocked for 1 h with PBS, 2% BSA (w/v) at 37 °C. Samples were diluted 1:50 in PBS, 2% BSA (w/v) and incubated for 60 min at 37 °C. Samples at the same dilution were also added to a non-coated well to assess individual nonspecific binding. After six further washes, 50 μl/well of alkaline phosphatase-conjugated anti-(human IgG) (Sigma), diluted 1:1000 in PBS, 2% BSA (w/v), was incubated for 1 h at 37 °C. After six more washes (150 μl/well) with PBS, 2% BSA (w/v), the phosphatase substrate *p*-nitrophenyl phosphate (50 μl/well; 1 mg/ml; Sigma) dissolved in 4.8% (w/v) diethanolamine, pH 9.8, was added. Each sample was tested in duplicate and *A*_405 nm_ was determined after 20 min of incubation at 37 °C (VERSAMax; Molecular Devices). The corresponding nonspecific binding was subtracted from the mean absorbance for each sample. For the competitive ELISA using commercial polyclonal antibodies against apoA-I (Academy Bio Medical and Agrisera), prior to the serum samples incubation, the apoA-I-coated and blocked plates were preincubated with different concentrations of the respective polyclonal antibody for 1 h at 37 °C. The cut-off value for positivity was prospectively defined and set at 0.64 optical density, as previously described (19, 45). Repeatability and reproducibility were determined at two levels. At a high level (*A*_405 nm_ = 1.2, *i.e.* twice the cut-off value), the intra- and interassay coefficients of variation were 10 (*n* = 10) and 17% (*n* = 10), respectively. At the cut-off level, the intra- and interassay coefficients of variation were 16 (*n* = 10) and 12% (*n* = 8), respectively.

##### ApoA-I Isolation and Purification by Thiophilic Interaction

Human derived apoA-I was purified and delipidated according to the described method (46) at a scale consistent with further purification using thiophilic interaction by covalent interaction on Activated Thiol-Sepharose 4B (GE Healthcare). This protocol takes advantage of the absence of cysteine residues in human apoA-I, so that apoA-I flows through the column, whereas other reduced thiol-containing proteins binds. Basically, 1 mg of apoA-I preparation was reduced in 5 mm DTT followed by buffer exchange in binding buffer (0.1 m Tris-HCl, pH 7.5, 0.5 m NaCl, 1 mm EDTA) using 10K filter (Amicon Ultra-0.5 10K, Millipore). Activated thiol-Sepharose (150 mg) was washed with a large excess of binding buffer, and then suspended in 1 ml of protein sample in binding buffer. This was incubated on a roller overnight at 4 °C. The flow through (non-thiol-containing proteins) was collected, and the thiol-containing proteins bound to the Sepharose were eluted by incubating with 1 ml of elution buffer (50 mm Tris-HCl, pH 8.0, 1 mm EDTA + 50 mm DTT) on a roller for 1 h at room temperature. Total protein concentration was determined by Bradford assay (47) and the specific apoA-I protein concentration was determined by ELISA and used to determine the purity of apoA-I after the purification steps. Proteins from different thiophilic interaction fractions were analyzed by SDS-PAGE and immunoblotting (Fig. 2).

##### Electrophoresis and Immunoblotting

Homogenates containing about 5 μg of apoA-I protein were heated for 5 min at 95 °C, and subjected to one-dimensional electrophoresis (SDS-PAGE) using NuPAGE 4–12% BisTris polyacrylamide gel on a Novex Mini-Cell (Invitrogen). After electrophoresis, the gels were submitted to silver staining, or the proteins were transferred from gel to a nitrocellulose membrane using the Trans-Blot SD semi-dry transfer cell (Bio-Rad). The nitrocellulose membranes were incubated with antibody specific to detect apoA-I (goat anti-human apoA-I, 11A-G2b, Academy BioMedical). Each membrane was incubated with compatible secondary antibodies conjugated with peroxidase, developed using Lumi-Light Western blotting Substrate Reagents (Roche Applied Science), and detection using x-ray equipment.

##### Enzymatic Digestion

To identify specific endogenous epitopes, the purified apoA-I was submitted to enzymatic digestion, followed by peptide separation and purification by reversed phase-high performance liquid chromatography and peptide identification by mass spectrometry. LysC hydrolyzes specifically at the carboxyl side of lysine, ArgC cleaves at the carboxyl side of arginine and trypsin cleaves peptide chains mainly at the carboxyl side of the amino acids lysine or arginine (supplemental Fig. S1). The immunoreactivity to the digested protein and each fraction were tested by ELISA using serum samples from 3 patients with high titers and serum samples from 3 patients with low titers of autoantibodies. Endoproteinase LysC (Roche Applied Science) was used at an enzyme/protein ratio = 1:50 by weight, with incubation for 18 h at 37 °C, pH 8.5. As ArgC has a recognized lack of specificity, apoA-I was reversibly blocked at lysine residues with maleic anhydride prior to digestion with trypsin (48, 49), generating a digestion cleaving specifically at arginine residues. After tryptic digestion, the maleyl group is removed by intramolecular catalysis at acid pH. Briefly, TIC-purified apoA-I (500 μg) was incubated with 5 μl of 33% (w/v) maleic acid anhydride dissolved in dioxin, adjusted to pH 9.0 with 4 m NaOH and a final volume to 200 μl with PBS, and incubated for 2 h at room temperature in the dark. Buffer was exchanged to 25 mm Tris-HCl and 1 mm EDTA, pH 8.5, by gel filtration, using PD Spin Trap G-25 columns. The apoA-I with Lys-maleyl residues was then submitted to tryptic digestion (enzyme/protein ratio = 1:50 by weight, incubation for 18 h at 37 °C). After digestion, the maleyl groups were removed by intramolecular catalysis at acid pH, adjusted with 1% (v/v) TFA, pH 3.5, and incubated for 24 h at 37 °C. To avoid precipitation, 5 m guanidine was added to the reaction. The unblocked peptides were then submitted to fractionation by RP-HPLC.

##### Peptide Fractionation by Reversed Phase-High Performance Liquid Chromatography

An aliquot (500 μl) containing 40 μg of digested apoA-I was injected on to the reversed phase-high performance liquid chromatography (RP-HPLC) system, consisting of a Shimadzu Model LC-10Ai pump set (Shimadzu) connected to a 7125 Rheodyne injector valve (Rheodyne). The peptides were fractionated with a 4.6 × 50-mm (particle size 5 μm) mRP Hi-Recovery Protein Column (Agilent), detected in a diode array detector (SPD-M20A, Shimadzu), and collected by an automatic collector liquid handler (Gilson). The mobile phases consisted of (A) 0.1% (v/v) TFA in water and (B) 0.1% (v/v) TFA in acetonitrile, at a flow rate of 0.5 ml/min, and using the following gradients: 1) digestion at lysine residues: 1–5 min, 10% buffer B: 5–60 min, 10–50% buffer B, 60–60.1 min, 50–80% buffer B; 60.1–63.1 min, 80% buffer B; or 2) digestion at arginine residues: 1–5 min, 10% buffer B; 5–60 min, 10–65% buffer B, 60–60.1 min, 65–90% buffer B; 60.1–63.1 min, 90% buffer B. Data acquisition was performed using the Shimadzu Class-VP chromatography data system software.

##### Peptide Identification by Mass Spectrometry

The LC-MS/MS measurements were performed on a LTQ-Orbitrap (Thermo Electron, Bremen, Germany) coupled to an Ultimate 3000 nano-flow chromatographer (Dionex, Germany). Peptides were separated on a 75 μm inner diameter × 15-cm length PicoTip nano-column (FS360–75-8-N-5-C20, New Objective) packed in-house with Reprosil-Pur C18-AQ (Dr. Maisch, Germany; particle size 3 μm). For analysis of the peptides derived from the digestion at lysine residues of apoA-I, mobile phases consisted of 0.5% acetic acid, 97.5% water, 2% acetonitrile (v/v/v) (A) and 0.5% acetic acid, 20% water in 79.5% acetonitrile (v/v/v) (B). In the case of peptides derived from the digestion at arginine residues of apoA-I, mobile phases consisted of 0.025% TFA, 97.975% water, and 2% acetonitrile (v/v/v) (A) and 0.025% TFA, 20% water in 79.975% acetonitrile (v/v/v) (B). Samples were diluted with mobile phase A to an appropriate concentration and 5 μl were directly loaded on-column at 450 nl/min for 13 min and analyzed using the following gradient: 1) digestion at lysine residues: 13–40 min, 0–20% buffer B at 250 nl/min; 40–55 min, 20–44% Buffer B; or 2) digestion at arginine residues: 13–40 min, 0–35% buffer B at 250 nl/min; 40–55 min, 35–59% buffer B. The column was then washed for 15 min with 100% buffer B at 350 nl/min and re-equilibrated for 29 min in 100% buffer A at 350 nl/min. Peptides were analyzed by tandem mass spectrometry using standard operating parameters as follows: the electrospray voltage was set to 2.0 kV (2.5 kV when using TFA) and the capillary temperature was at 170 °C. Survey scans (scanning range *m*/*z* 400–1650) were recorded in the Orbitrap mass analyzer at a resolution of 60,000 with the lock mass option enabled. Data-dependent MS/MS spectra of the three most abundant ions (threshold of 500) from the survey scan were performed in the LTQ ion trap using a normalized collision energy of 35% for MS/MS (30 ms activation, *q* = 0.25) to be then recorded in the Orbitrap mass analyzer at a resolution of 15,000 with the lock mass option enabled. Target ions selected for MS/MS were dynamically excluded for 30 s. Raw data were processed using the SEQUEST search algorithm 27 (SEQUEST version 27.0, revision 12, Thermo Electron). Total digests were searched against the Swiss-Prot database (filtered for the human taxonomy; 35,106 entries) considering only tryptic peptides. HPLC-purified peptides were searched against a database consisting of the human apoA-I sequence with no enzyme specificity considered. Data were searched with a mass tolerance of ±5 ppm for parent ions and 50 mm units for fragment ions. Methionine sulfoxide (+15.9949 Da) and lysine carbamylation (+43.005814 Da) were considered as differential modification throughout. For the fragments submitted to reaction with maleic anhydride, maleimide-derivatized lysines (+97.016378 Da) were considered to take into account the incomplete unblocking of the lysine residues after digestion at arginine residues. All considered hits were manually examined to confirm the sequence assignment.

##### Synthetic Peptides

The apoA-I synthetic peptides, amino acids 165–206, LSPLGEEMRDRARAHVDALRTHLAPYSDELRQRLAARLEALK, and oxidized analog, LSPLGEEM(O)RDRARAHVDALRTHLAPYSDELRQRLAARLEALK, and the C-terminal peptide amino acid 241–266, GLLPVLESFKVSFLSALEEYTKKLNT, were synthesized by standard Fmoc protocol on a Wang resin using a PTI-Prelude apparatus (Protein Technologies). M(O) was incorporated as the Fmoc-protected methionine sulfoxide. Peptides were cleaved from the resin with 90% trifluoroacetic acid, 5% phenol, 2.5% water, and 2.5% triisopropylsilane, precipitated in diethyl ether, purified via reversed-phase HPLC, and lyophilized. The expected masses for each peptide were checked by mass spectrometry (LTQ-Orbitrap).

## RESULTS

### 

#### 

##### Determination of Antigen Specificity of ApoA-I by Competitive ELISA

**FIGURE 1.**
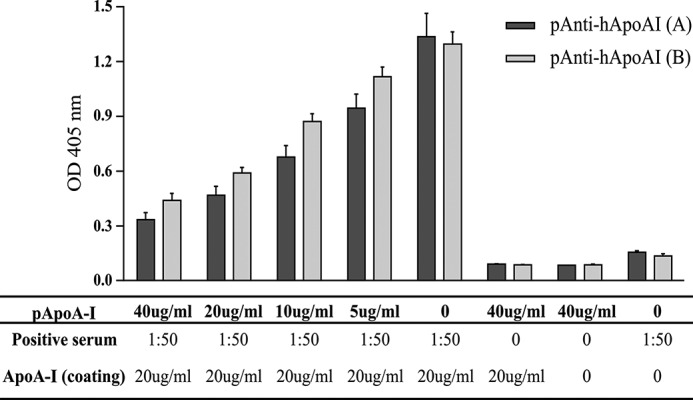
**Antigen specificity for human apoA-I by competitive ELISA.** Commercial polyclonal anti-apoA-I antibodies: Academy BioMedical (*A*) and Agrisera (*B*), compete for apoA-I binding sites with the autoantibodies from patient serum. Three independent experiments were performed. Positive serum: samples previously classified as containing high titers of anti-apoA-I IgG.

The standard extraction of apoA-I from plasma by HDL fractionation, delipidation, and size exclusion chromatography typically yields protein about 95% pure (46). This apoA-I preparation was coated on the immunoassay plate and probed with serum as the source of autoantibody. As other immunoreactivity proteins could be present, a competitive ELISA was used to evaluate the specificity of the binding antigen/antibody. Two different commercial polyclonal antibodies against human apoA-I were able to compete with autoantibodies present in the serum from a patient previously classified as containing high titer anti-apoA-I IgG (Fig. 1).

##### Isolation and Purification of Human Apolipoprotein A-I

**FIGURE 2.**
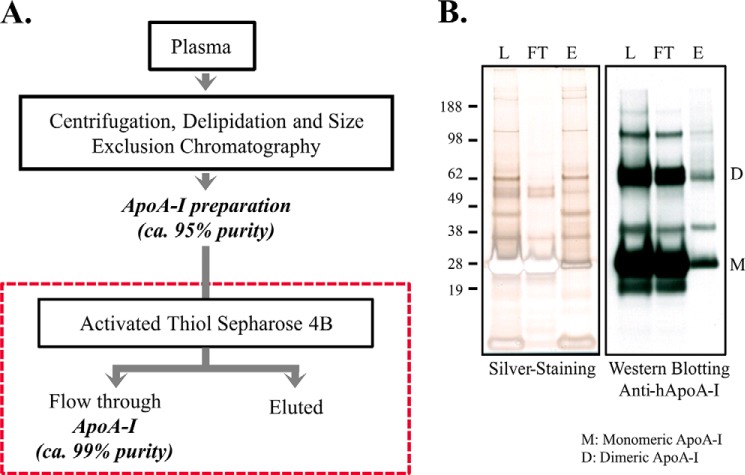
**ApoA-I purification from human plasma.** *A*, scheme showing steps involved in apoA-I purification process, including thiophilic interaction using activated thiol-Sepharose 4B. *B*, silver staining electrophoresis gel and immunoblotting of thiophilic interactions fractions. *L,* loading; *FT*, flow-through; *E,* eluted.

To identify the endogenous epitope recognized by anti-apoA-I IgGs, we created a higher purity human apoA-I preparation. ApoA-I was purified from plasma by centrifugation, delipidation, and size exclusion chromatography at a scale consistent with further purification and characterization of the epitope. Because apoA-I has no cysteine in its sequence, we further purified the isolated apoA-I (about 95% purity) by TIC, selecting the apoA-I monomer/oligomers from other thiol-containing proteins. In this protocol, apoA-I appeared in the flow through, whereas reduced thiol-containing biomolecules bonded to the column. This generated a fraction containing ∼99% pure apoA-I as observed by electrophoresis and immunoblotting (Fig. 2) and by total and apoA-I protein quantification (data not shown). A high level of immunoreactivity to the high purity apoA-I was detected by ELISA, using patient samples containing high titers of autoantibodies (Table 1).

**TABLE 1:** **Immunoreactivity of thiophilic interaction fractions**

Solid phase	Positive serum*^a^*			Negative serum*^a^*		
	Avg.*^b^*	S.D.	CV	Avg.	S.D.	CV
*20* μ*g/ml*						
**ApoA-I preparatioin (loading)**	1.248	0.106	8.5	0.209	0.004	1.8
**Flow-through (FT)*^c^***	1.124	0.061	5.5	0.164	0.040	24.3
**Eluted (E)**	0.242	0.014	5.8	0.113	0.003	2.6
**Blank**	0.171	0.011	6.4	0.106	0.001	1.1

*^a^* Positive serum: samples previously classified as containing high titers of anti-apoA-I IgG; negative serum: samples previously classified as containing low titers of anti-apoA-I IgG.

*^b^* Average of the optical density at 405 nm (arbitrary unit) readout from three individual ELISA measurements.

*^c^* ApoA-I containing thiophilic interaction fraction. Serum dilution: 1:50.

##### Human ApoA-I Enzymatic Digestion, Peptide Isolation, and Identification by Mass Spectrometry

The high purity apoA-I isolated through TIC was enzymatically digested using different enzymes (LysC, ArgC, and trypsin). The different peptides generated were then tested in the ELISA, probing for immunoreactivity of the peptides with 3 serum samples derived from myocardial infarction patients containing high levels of anti-apoA-I IgG and 3 serum samples from patients with low levels of anti-apoA-I antibodies.

**FIGURE 3.**
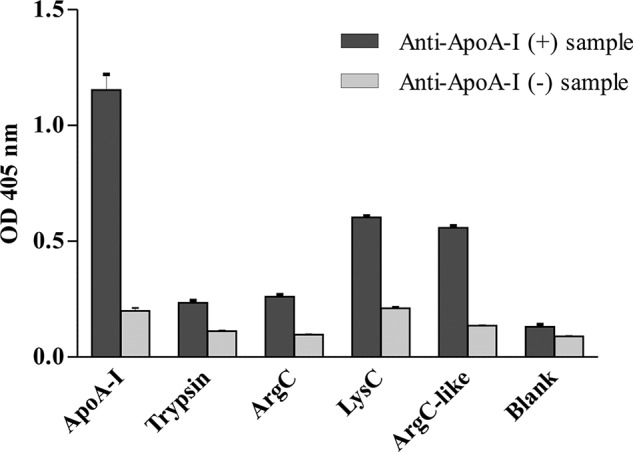
**Immunoreactivity of full-length apoA-I and digested extracts.** The graph shows the levels of immunoreactivity (OD at 405 nm, arbitrary unit) of autoantibodies from a sample previously classified as high (+) or low (−) titer of anti-apoA-I IgG against the full-length human apoA-I and digested extracts by several enzymes: trypsin (cleaving C-terminal of Lys and Arg), ArgC (theoretically, cleaving C-terminal of Arg), LysC (cleaving C-terminal of Lys), and ArgC-like (cleavage using trypsin after blocking of lysine groups).

The digested apoA-I using trypsin or ArgC did not retrieve any immunoreactivity. We considered that the generation of small peptides by trypsin was likely to have destroyed potential epitopes. We rationalized that larger fragments generated by the enzyme LysC would create larger peptides and increase the probability of maintaining secondary structure integrity, and also antigenicity (50). We confirmed by mass spectrometry that ArgC digestion lacked specificity, digesting at both arginine and lysine residues. We therefore performed a digestion with trypsin, where the lysine residues were protected to create specific cleavage N-terminal of arginine residues (ArgC-like digestion). Fig. 3 illustrates that immunoreactivity was observed following either LysC- or ArgC-like digestion. The peptides generated by enzymatic digestions were fractionated using RP-HPLC. For each individual fraction containing isolated digested peptides, we tested the immunoreactivity by ELISA using 3 serum samples from cardiac patients with high titers and 3 serum samples from cardiac patients with low titers of autoantibodies against apoA-I. During the peptide fractionation by HPLC, a potential issue is the presence of acetonitrile interfering with the immunoreactivity. However, this was not observed and the potential immunoreactivity from the full-length apoA-I was positive even after exposure to acetonitrile (supplemental Fig. S2).

**FIGURE 4.**
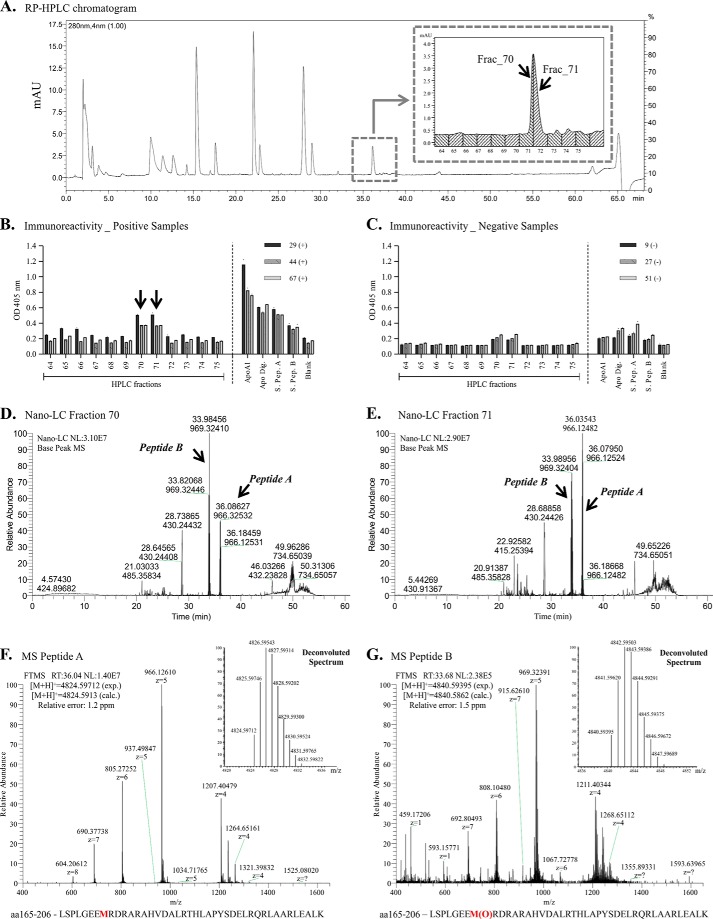
**ApoA-I epitope identification using digestion at lysine residues.** *A*, chromatogram of LysC-derived peptides fractionation by reversed phase-high performance liquid chromatography. *B* and *C*, immunoreactivity of autoantibodies from positive patient sera (*B*) and negative patient sera (*C*) against peptides present in each collected fraction, and against the synthetic peptides A and B. *D* and *E,* chromatogram of the immunoreactive fraction 70 (*D*) and fraction 71 (*E*) by nano-liquid chromatography system. *F* and *G*, representative mass spectra of the peptide A (*F*) and peptide B (*G*) present in immunoreactive fractions by mass spectrometry (LTQ/Orbitrap). Relative error = (M_experimental_ − M_calculated_)/M_calculated_. Positive or negative sera: samples previously classified as containing high-titers or low titers of anti-apoA-I IgG, respectively.

Endogenous apoA-I submitted to LysC digestion generated two main fractions showing immunoreactivity (70 and 71) using samples containing autoantibodies against apoA-I (Fig. 4, *A* and *B*). The analysis also showed lower immunoreactivity using samples previously classified as low titers for autoantibodies against apoA-I (Fig. 4*C*). In fact, these two fractions correspond to the same chromatographic peak (Fig. 4*A, inset*).

Tandem mass spectrometric analyses of those two main immunoreactive fractions (Fig. 4, *D* and *E*) identified two possible and related epitopes: Peptide A (Leu^165^-Lys^206^ [M + 5H]^5+^ = 965.7257, [M + H]^+^ = 4824.5971, relative error: 1.2 ppm) and Peptide B (Leu^165^-Lys^206^ with Met^172^ oxidized, [M + 5H]^5+^ = 968.9224, [M + H]^+^ = 4840.5939, relative error: 1.5 ppm). The tandem MS spectra for peptides A and B, as well as the deconvoluted spectra, are shown in Fig. 4, *F* and *G,* respectively. These fragments both represent a peptide sequence localized in one of the α-helices of apoA-I with peptide B bearing an oxidized methionine. Both are highly hydrophilic, presenting seven arginine residues, which confer to the peptides high charge when analyzed by mass spectrometry (Fig. 4, *F* and *G*, and supplemental Fig. S3). The peptides were observed to have a tendency to aggregate during the chromatographic run, complicating the separation and resolution. To overcome this issue, the mobile phase used in the nano-LC coupled to the mass spectrometer was changed from acetic acid to trifluoroacetic acid. Supplemental Fig. S3C shows the extracted ion chromatogram specifically of peptides A and B in the isolated fractions 70 and 71 from apoA-I digested at lysine residues. Information about the sequencing and MS/MS spectra of peptides A and B is available in supplemental Fig. S3, *A* and *B*.

Although the nano-LC system coupled to mass spectrometry resolved and separated peptides A and B, they eluted from the RP-HPLC at the same retention time using a preparative C18 column, hampering definition of which peptide(s) is responsible for the observed immunoreactivity. To overcome this issue we synthesized both peptides and tested the immunoreactivity using samples from patients previously classified as high titers for anti-apoA-I IgG. Fig. 4, *B* and *C,* indicates that both peptides showed the same level of immunoreactivity, indicating that the presence of the oxidized methionine may not be essential for the antigen immunogenicity of this specific peptide.

**FIGURE 5.**
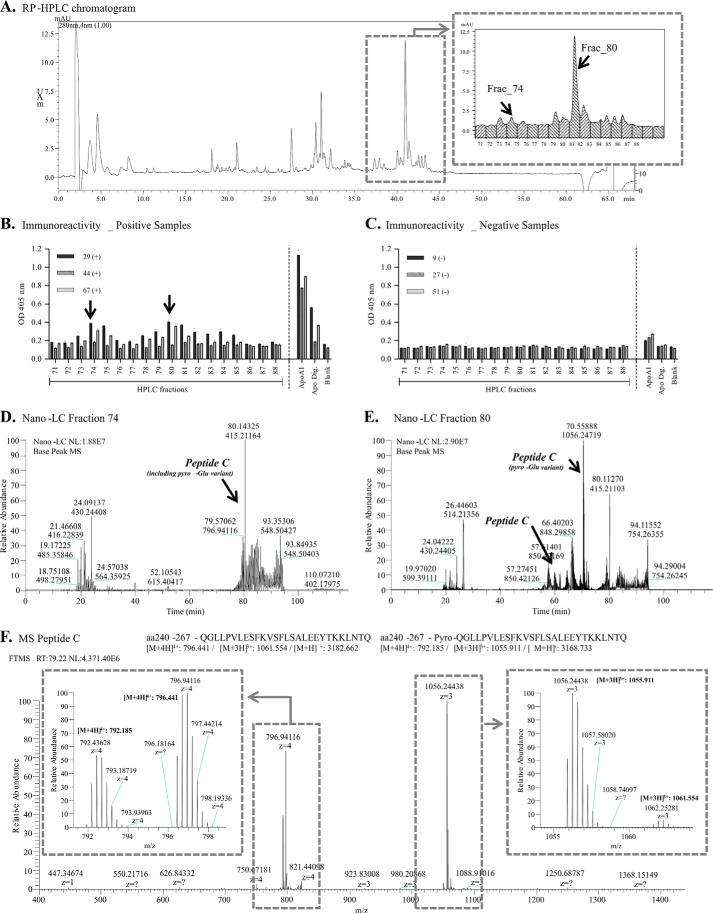
**ApoA-I epitope identification using digestion at arginine residues.** *A*, chromatogram of peptides separation by reversed phase-high performance liquid chromatography. *B* and *C,* immunoreactivity of autoantibodies from positive patient sera (*B*) and negative patient sera (*C*) against peptides present in each collected fraction, and against the synthetic peptide. *D* and *E*, full chromatograms of the immunoreactive fraction 74 (*D*) and fraction 80 (*E*) by nano-liquid chromatography system. *F*, representative mass spectrum of peptide C and its pyro-Glu variant present in the immunoreactive fraction 74 by mass spectrometry (LTQ/Orbitrap). Relative error = (M_experimental_ − M_calculated_)/M_calculated_. Positive or negative sera: samples previously classified as containing high titers or low titers of anti-apoA-I IgG, respectively.

To circumvent the ArgC lack of specificity generating tryptic peptides, we used reversible blocking of lysine residues with maleic anhydride followed by tryptic digestion and followed by subsequent unblocking under acid pH (49) to generate fragments cleaved at the N-terminal of arginine residues. The fragments obtained by this methodology were separated by HPLC (Fig. 5*A*). Several fractions, which eluted between retention times of 37 to 44 min, showed immunoreactivity (threshold 1.5 above to the blank value) using samples previously classified as containing high titer of anti-apoA-I autoantibodies (Fig. 5*B*), whereas samples previously classified as low titer showed low immunoreactivity (Fig. 5*C*). The mass spectrometric analysis for fractions 74 and 80 are shown in Fig. 5, *D* and *E,* respectively. In these fractions, the peptide Gln^240^-Gln^26^Q ([M + H]^+^ = 3182.7347, Peptide C) and its N-terminal pyroglutamic variant ([M + H]^+^ = 3165.7082) were identified by tandem mass spectrometry (Fig. 5*F* and supplemental Fig. S4). The pyroglutamate group is formed when the free amino group of glutamic acid or glutamine cyclizes spontaneously during isolation and proteolysis and as such is likely to be an artifact of the analysis (51). The peptides appeared as triple and quadruple charges (Fig. 5*F* and supplemental Fig. S5); and as expected, the pyroglutamic variant was detected with a high level of triple charge ([M + H]^3+^ = 1055.9105, relative error: 2.7 ppm), whereas natural variant was detected predominantly as the quadruple charge ([M + H]^4+^ = 796.4407, relative error: 5.3 ppm). Most importantly, the apoA-I synthetic peptide Gly^241^-Thr^266^ was immunoreactive when exposed to samples from patients with high titers of autoantibodies against apoA-I (Fig. 5*B*), reinforcing our findings using endogenous fragments and corroborating the potential presence of an epitope in the C-terminal region of apoA-I.

## DISCUSSION

Recent studies demonstrated that IgG autoantibodies against apoA-I are raised in many diseases associated with a high cardiovascular risk, such as systemic lupus erythematosus, acute coronary syndrome, myocardial infarction, rheumatoid arthritis, severe carotid stenosis, and end-stage renal disease (11, 12). However, identifying whether the immune response to apoA-I could be focused on a specific part of apoA-I still remained elusive. In this study, we identified two epitopes, both localized on helices of apoA-I, which are recognized by anti-apoA-I autoantibodies derived from patients with myocardial infarction. Thus, the present results lend weight to hypothesis that the anti-apoA-I autoimmune response observed in CVD patients could well be directed to specific parts of apoA-I despite its polyclonal nature. Although the present results indicate that such apoA-I epitopes could represent new markers for prediction of CVD further work with clinically relevant cohorts will be needed to establish this. Nevertheless, our data are consistent with several studies suggesting the possibility that markers of immune response to antigens raised in the atherosclerotic lesions could be used to determine disease activity and predict the risk of development of acute cardiovascular events (11, 52–54). Furthermore, because *in vitro* and *in vivo* studies published so far indicated a direct pro-inflammatory role of those autoantibodies (16–18, 20), we speculate that the corresponding synthetic peptides could be engineered and used to block the deleterious effects of those autoantibodies. This hypothesis is currently being tested in our laboratory.

The generation of high purity apoA-I (99%) obtained presently through TIC was an important hurdle to overcome to define the specificity of anti-apoA-I antibodies, limiting interferences from possible contaminants when using less pure antigenic source. Currently, the results using competitive ELISA and highly pure apoA-I corroborate with the hypothesis of the presence of autoantibodies specific for apoA-I and suggest two different epitopes located on the α-helical regions of apoA-I in samples from patients with CVD.

Furthermore, the fact that tryptic digestion of human apoA-I completely abrogated autoantibody immunoreactivity lends weight to the hypothesis that the epitopes identified presently may be conformational. Indeed, a decreased reactivity to short linear synthetic peptides when compared with immunoreactivity observed against the intact molecule suggest that such peptides are fractions of a complete conformational epitope (50), often consisting of 15–22 amino acid residues in contact with the combining site of the antibody, and a subset of critical contact residues that contribute most of the free binding energy (55). The digestion of apoA-I at lysine or arginine residues generates fragments between 15 and 42 residues in length, which are more likely to isolate intact epitopes. One of the peptides isolated through digestion at lysine residues of apoA-I comprised a sequence of 42 residues (165–206 amino acids) and included one of the apoA-I α-helices. This sequence is highly hydrophilic, containing 6 arginine residues. Interestingly, Dohlman *et al.* (57) examined the autoantigen repertoire for structural properties that are known to promote antigenicity in a foreign protein, such as multivalency, repetitive motifs, a high content of charged or aromatic residues (56). They found that human target autoantigens often have charged surfaces, and have a high frequency of coiled-coils, a pair of α-helices that interact along their length and have a highly charged surface with repetitive elements, compared with a random selection of other protein sequences in GenBank^TM^ (57). Similarly, autoantigens associated with systemic lupus erythematosus are characterized by supercharged surfaces that render the targeted host proteins strongly immunogenic properties (58). The peptide Leu^165^-Lys^206^ includes a methionine at position 172 (148 at the mature apoA-I), which lies in the lecithin:cholesterol acyltransferase activation domain of apoA-I (59). We also identified the version of this peptide with the oxidized methionine in the immunoreactive HPLC fraction. Nevertheless, according to our results using synthetic peptides, it seems that the presence of the oxidized methionine is not required for immunoreactivity of anti-apoA-I autoantibodies. In fact, the presence of the oxidation slightly (but not statistically significantly) reduced the OD value as compared with the unmodified peptide. However, it is important to stress that oxidation of methionine residues could affect the structure and stability of apoA-I (60), exposing/creating new antigens. Moreover, the oxidation of the Met^172^ impairs the ability of apoA-I to activate lecithin:cholesterol acyltransferase, affecting the reverse cholesterol transport in the artery wall. Also, the presence of anti-apoA-I IgG in autoimmune disease has been associated with dysfunctional HDLs (61, 62), which could be generated by myeloperoxidase-catalyzed apoA-I oxidation (63, 64). Interestingly, although no correlation between the levels of anti-apoA-I and myeloperoxidase were retrieved in a study using samples from patients undergoing carotid endarectomy, combining the assessment of anti-apoA-I and myeloperoxidase provided incremental major adverse cardiovascular events predictive ability over Framingham risk score (65).

The selective enzymatic digestion of apoA-I at arginine residues was possible due to the reversible blocking of lysine residues with maleic anhydride followed by tryptic digestion. After the blocking reaction, maleyl proteins have an increased negative charge at neutral pH minimizing protein-protein interactions and protein-water interactions are favored (49). This improves the enzymatic digestion at arginine residues of apoA-I. This procedure allowed the generation of new peptides and overcame the issue related to nonspecificity of the enzyme ArgC. The unblocking of the lysine residues at acid pH was essential for further testing the immunoreactivity of the autoantibodies. Not only could lysine modifications create new epitopes or abrogate a possible immunoreactivity, but effects on helical content, lipid binding, and cholesterol acceptor activity have already been described due to apoA-I lysine modifications (66). Using this procedure, we could isolate the peptide Gln^240^-Gln^267^, and its pyroglutamic variant derived from the spontaneous cyclization of the free amino group of glutamine, which shows immunoreactivity when tested with samples from patients with high titers of autoantibodies against apoA-I. This peptide comprises the last α-helix domain of apoA-I. The C-terminal helix (residues 247–267 or 223–243 in the mature apoA-I) is a non-polar segment of the human apoA-I molecule and its hydrophobicity plays a crucial role in apoA-I to solubilize lipids and promote cholesterol efflux (67). The detection of immunoreactivity to the synthetic peptide Gly^241^-Thr^266^ when exposed to samples from patients with high titers of autoantibodies against apoA-I reinforces the potential presence of an epitope in the C-terminal region of apoA-I. In a parallel approach followed by our group using mimetic peptides from apoA-I, a similar peptide also covering the C-terminal helix and structurally stabilized by a lactam bridge was identified. This peptide also presented immunoreactivity when tested with samples with high titers of anti-apoA-I IgG. Competitive ELISA shows that this mimetic peptide competes effectively with intact apoA-I for binding to anti-apoA-I antibodies.

We are aware of some limitations of the current study. First of all, due to the limited amount of patient material, we could not formally demonstrate the pathology-associated effects of the samples used in the present study. Nevertheless, given the highly reproducible pro-inflammatory and pro-arythmogenic effects of anti-apoA-I IgG *in vitro* previously demonstrated (16–18, 68), we expect the anti-apoA-I positive samples used in this study to behave in pro-inflammatory and pro-arythmogenic ways *in vitro*. Further epitope mapping and specific apoA-I autoantigen characterization will be paramount to clarify the potential of autoantibodies as diagnostic tools, or even as therapeutic targets. It is possible that use of LysC- and ArgC-like digestion may have destroyed other potential epitopes. The enzymatic digestion may also destroy the secondary conformation that is important for antigenicity. One other important aspect to be considered is the possibility that the experimental procedures and chemical reactions have created a new epitope, which could cause some artifacts on the assay. Moreover, further analysis of the individual peptides, as well as its sequence refinement, may lead to better definition of the epitopes. Whether specific immunoreactivity to those fragments could recapitulate the prognostic value of immunoreactivity to the whole apoA-I molecule remains to be shown. Nevertheless, once identified, the pathogenic role of these epitopes can now be evaluated and potentially provide reagents that could have important diagnostic and therapeutic applications.

In conclusion, our epitope mapping methodology using antigen purification and enzymatic digestion successfully identified new epitopes recognized by autoantibodies in samples from patients previously classified as high titer for anti-apoA-I IgG. Of interest, ∼20% of patients with cardiovascular diseases do not display any Framingham risk factors (69), illustrating the importance of identifying new and reversible emergent CVD risk factors. Because antibody-mediated diseases and some cardiovascular conditions can be treated by specific immunologic therapeutic strategies, such as passive immunization (70), autoantibodies against apoA-I could represent an innovative therapeutic candidate. Moreover, the identification of a subset of CVD that could benefit from this kind of therapy and substantially contribute to personalized healthcare in the field of CVD, and therefore, highlighting the importance of specific epitope definition.

## Supplementary Material

Supplemental Data

## Footnotes

3 The abbreviations used are:

CVD: cardiovascular disease
apoA-I: apolipoprotein A-I
RP: reversed phase
TIC: thiophilic interaction chromatography
BisTris: 2-[bis(2-hydroxyethyl)amino]-2-(hydroxymethyl)propane-1,3-diol
Fmoc: *N*-(9-fluorenyl)methoxycarbonyl.
