# Supplementary material for: Definition of Human Apolipoprotein A-I Epitopes Recognized by Autoantibodies Present in Patients with Cardiovascular Diseases
Source: J Biol Chem. 2014 Aug 28;289(41):28249–59. doi: 10.1074/jbc.M114.589002 (PMC4192480; doi:10.1074/jbc.M114.589002)
Supplement: Supplemental Data [file supp_289_41_28249__index.html]

Definition of human apolipoprotein A-I epitopes recognized by autoantibodies present in patients with cardiovascular diseases — Definition of Human Apolipoprotein A-I Epitopes Recognized by Autoantibodies Present in Patients with Cardiovascular Diseases — Immunogenic Peptides in Human ApoA-I — Supplemental Data 

# Definition of Human Apolipoprotein A-I Epitopes Recognized by Autoantibodies Present in Patients with Cardiovascular Diseases

## Supplemental Data

**Files in this Data Supplement:**

- Supporting information (.pdf, 1.4 MB) - This file contain supplemental results that support the manuscript.
