# Supplementary material for: Definition of Human Apolipoprotein A-I Epitopes Recognized by Autoantibodies Present in Patients with Cardiovascular Diseases
Source: J Biol Chem. 2014 Aug 28;289(41):28249–59. doi: 10.1074/jbc.M114.589002 (PMC4192480; doi:10.1074/jbc.M114.589002)
Supplement: Supplemental Data [file supp_M114.589002_jbc.M114.589002-1.pdf]

Definition of human apolipoprotein A-I epitopes recognized by autoantibodies present in patients with cardiovascular diseases.

Supporting Information

Figure S1. APOA1_HUMAN protein sequence.

APOA1_Human protein sequence

10	20	30	40	50	60
MKAAVLTLAV	LFLTGSQARH	FWQQDEPPQS	PWDRVKDLAT	VYVDVLKDSG	RDYVSQFEGS
70	80	90	100	110	120
ALGKQLNLKL	LDNWDSVTST	FSKLREQLGP	VTQEFWDNLE	KETEGLRQEM	SKDLEEVKAK
130	140	150	160	170	180
VQPYLDDFQK	KWQEEMELYR	QKVEPLRAEL	QEGARQKLHE	LQEKLSPLGE	EMRDRARAHV
190	200	210	220	230	240
DALRTHLAPY	SDELRQRLAA	RLEALKENGG	ARLAEYHAKA	TEHLSTLSEK	AKPALEDLRQ
250	260				
GLLPVLESFK	VSFLSALEEY	TKKLNTQ			

Cleavage at Lysine (K) residues

mass	position	peptide sequence
4824.59	165-206	LSPLGEEMRDRARAHVDALRTHLAPYSDELRQRLAARLEALK
2202.12	84-101	LREQLGPVTQEFWDNLEK
2025.14	233-250	PALEDLRQGLLPVLESFK
1815.85	48-64	DSGRDYVSQFEGSALGK
1723.94	143-157	VEPLRAELQEGARQK
1612.79	70-83	LLDNWDSVTSTFSK
1539.73	132-142	WQEEMELYRQK
1453.71	25-36	DEPPQSPWDRVK
1415.70	207-219	ENGGARLAEYHAK
1386.72	251-262	VSFLSALEEYTK
1307.63	102-112	ETEGLRQEMSK
1252.62	121-130	VQPYLDDFQK
1235.69	37-47	DLATVYVDVLK
1215.62	220-230	ATEHLSTLSEK
896.48	158-164	LHELQEK
732.38	113-118	DLEEVK
615.38	65-69	QLNLK

Cleavage at Arginine (R) residues

mass	position	peptide sequence
4161.03	108-140	QEMSKDLEEVKAKVQPYLDDFQKKWQEEMELYR
3859.99	52-85	DYVSQFEGSALGKQLNLKLLDNWDSVTSTFSKLR
3182.73	240-267	QGLLPVLESFKVSFLSALEEYTKKLNTQ
3021.60	213-239	LAEYHAKATEHLSTLSEKAKPALEDLR
2618.27	86-107	EQLGPVTQEFWDNLEKETEGLR
2165.14	156-173	QKLHELQEKLSPLGEEMR
1878.03	35-51	VKDLATVYVDVLKDSGR
1301.65	185-195	THLAPYSDELR
1226.54	25-34	DEPPQSPWDR
1157.63	202-212	LEALKENGGAR
873.44	148-155	AELQEGAR
869.52	141-147	QKVEPLR
781.43	178-184	AHVDALR

Figure S1. APOA1_HUMAN protein sequence. Human ApoA-I protein sequence (APOA1_HUMAN / P02647) and peptides derived from the cleavage at lysine or arginine residues.

Figure S2. Human ApoA-I fractionation.

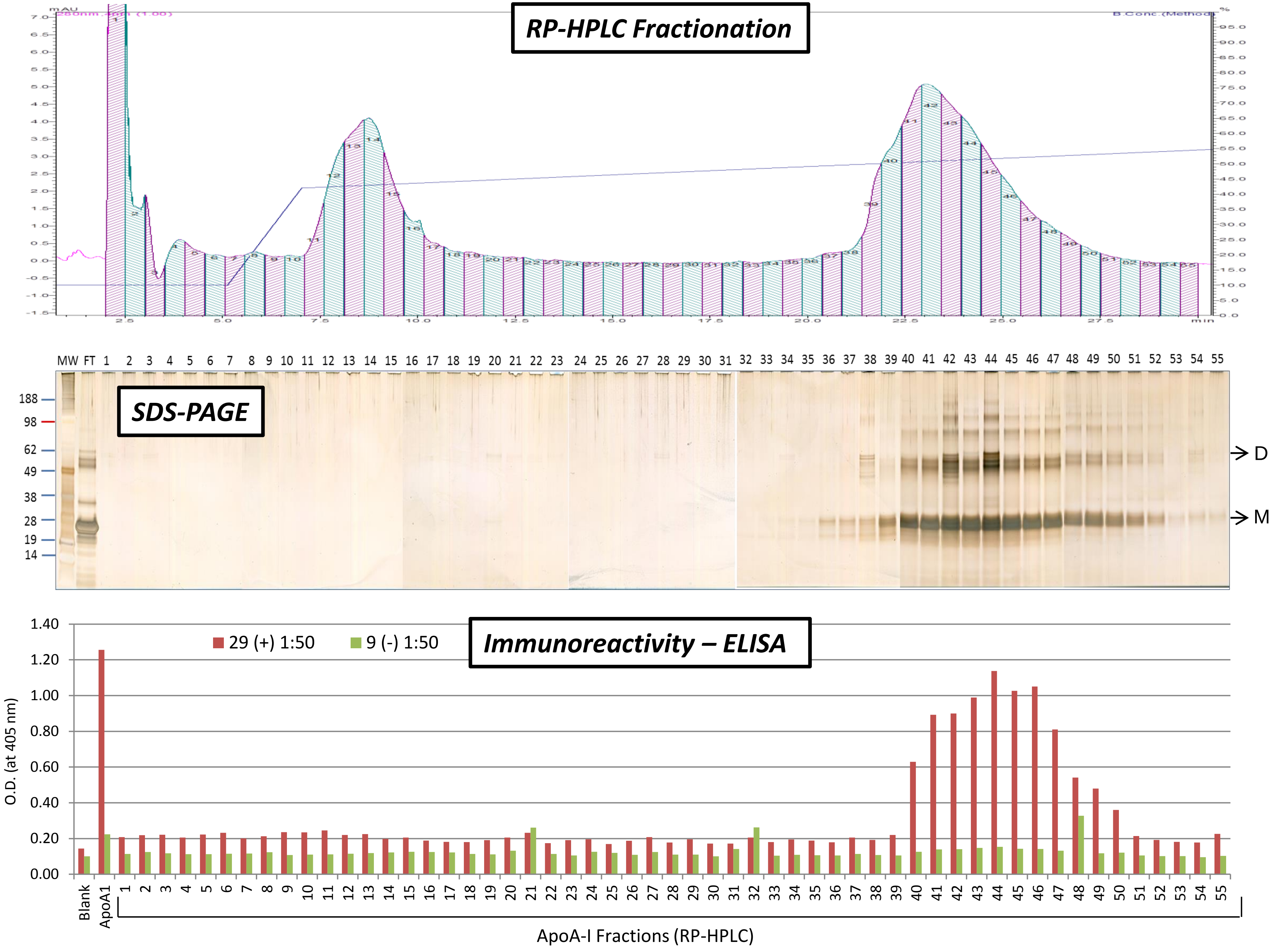

RP-HPLC Fractionation
mAU
280nm,4nm (1.00)
B.Conc.(Method)
%
min
SDS-PAGE
MW FT
188
98
62
49
38
28
19
14
D
M
Immunoreactivity – ELISA
29 (+) 1:50
9 (-) 1:50
O.D. (at 405 nm)
Blank
ApoA1
ApoA-I Fractions (RP-HPLC)

Figure S2. Human ApoA-I fractionation.

A. Chromatogram of full length human ApoA-I separation by reversed-phase high-performance liquid chromatography, emphasizing the collected fractions.

B. Electrophoresis (SDS-PAGE, silver-staining) of each individual fraction collected during the ApoA-I fractionation (D: dimer, M: monomer).

C. Immunoreactivity of autoantibodies from positive and negative patient sera against peptides present in each collected fraction.

Figure S3. Information about the sequencing and MS/MS spectra of the peptide A and B obtained by ApoA-I digestion at lysine residues.

A. MS/MS - Fraction 70/71: m/z 966.13 (5+); RT: 36.20 min – Peptide A

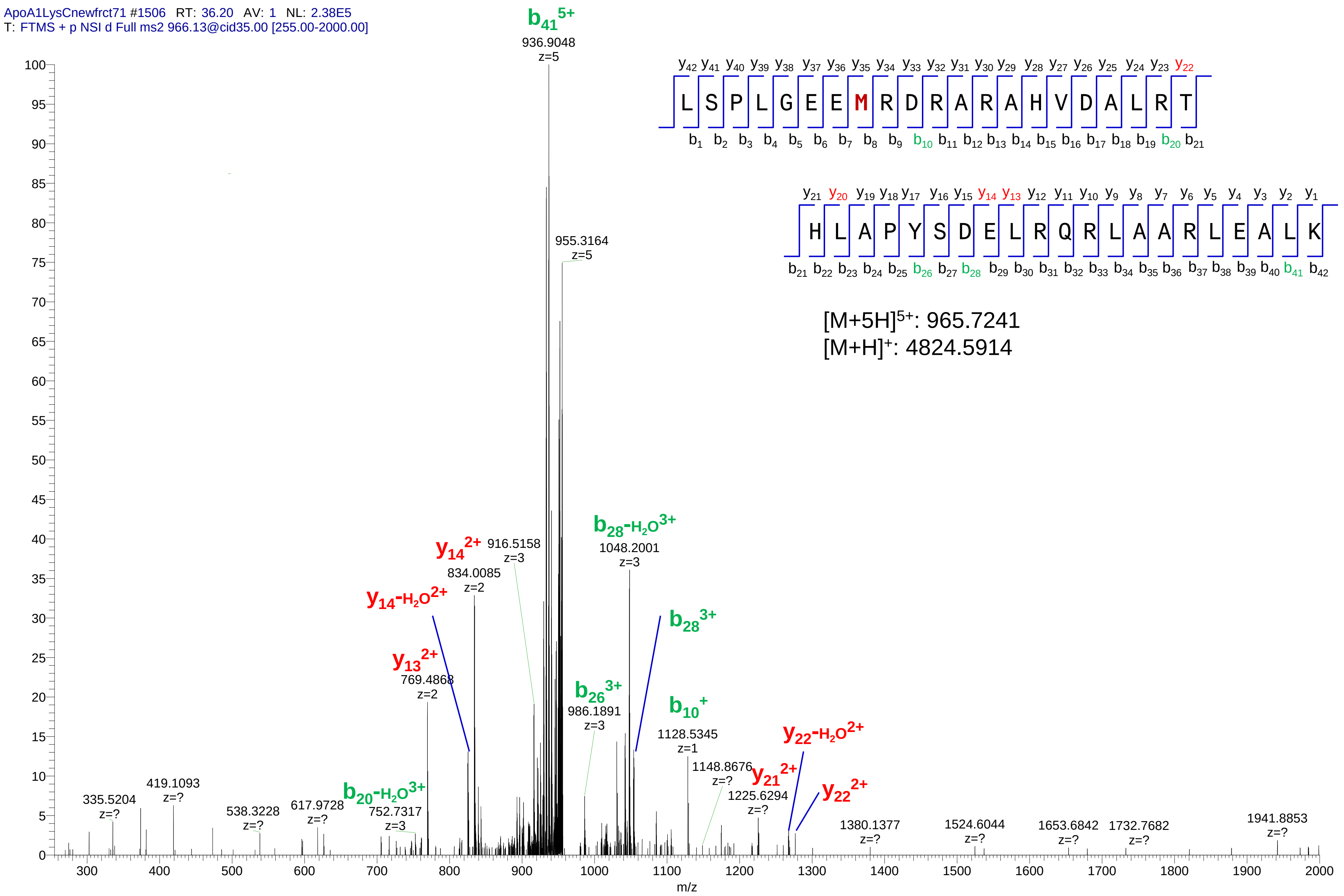

B. MS/MS - Fraction 70/71: m/z 969.32 (5+); RT: 33.69 min – Peptide B

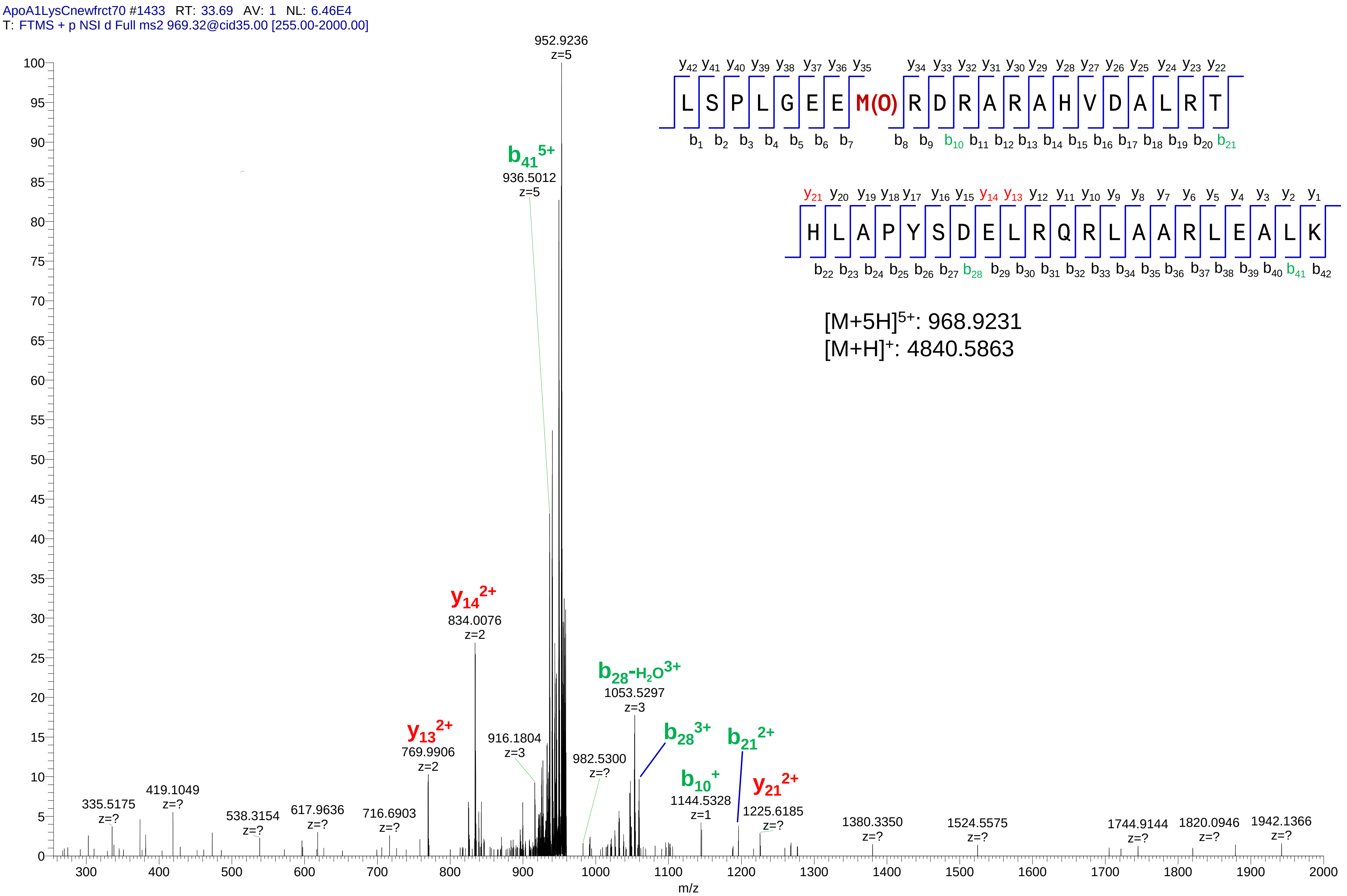

C. Extracted Ion Chromatogram of the peptides A and B in isolated fractions from ApoA-I digested at lysine residues.

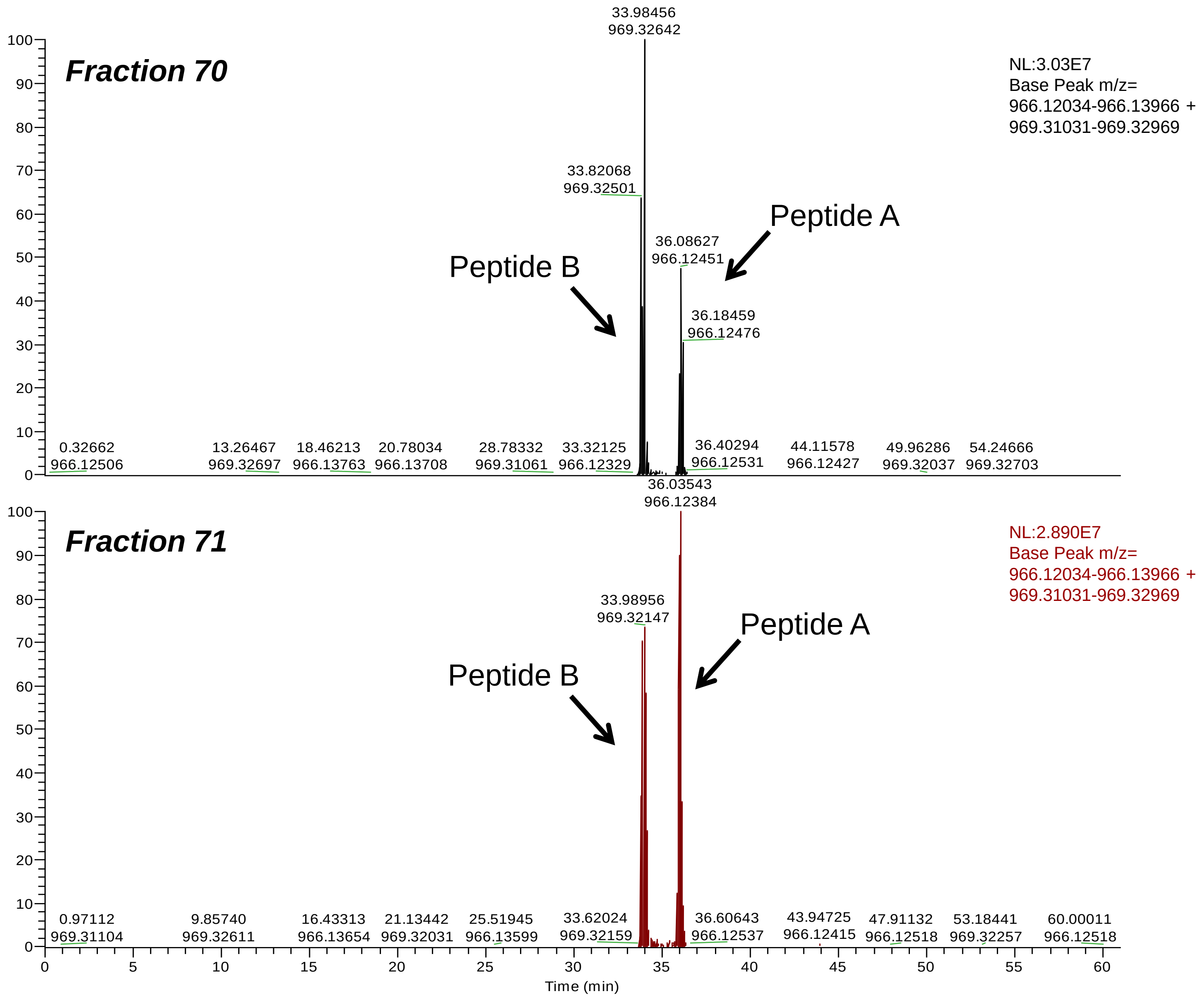

Figure S3. Information about the sequencing and MS/MS spectra of the peptide A and B obtained by ApoA-I digestion at lysine residues.

A. Representative MS/MS spectra of the peptide A present in the fractions 70 and 71: aa165-206 – LSPLGEEMRDRARAHVDALRTHLAPYSDELRQRLAARLEALK.

B. Representative MS/MS spectra of the peptide A present in the fractions 70 and 71: aa165-206 – LSPLGEEM*RDRARAHVDALRTHLAPYSDELRQRLAARLEALK; *Oxidized methionine.

C. Extracted ion chromatogram of the peptides A and B.

Figure S4. ApoA-I epitope identification using digestion at arginine (R) residues.

A. Full chromatograms (nano-LC) of isolated fractions from ApoA-I digested at arginine (R) residues (Part 1)

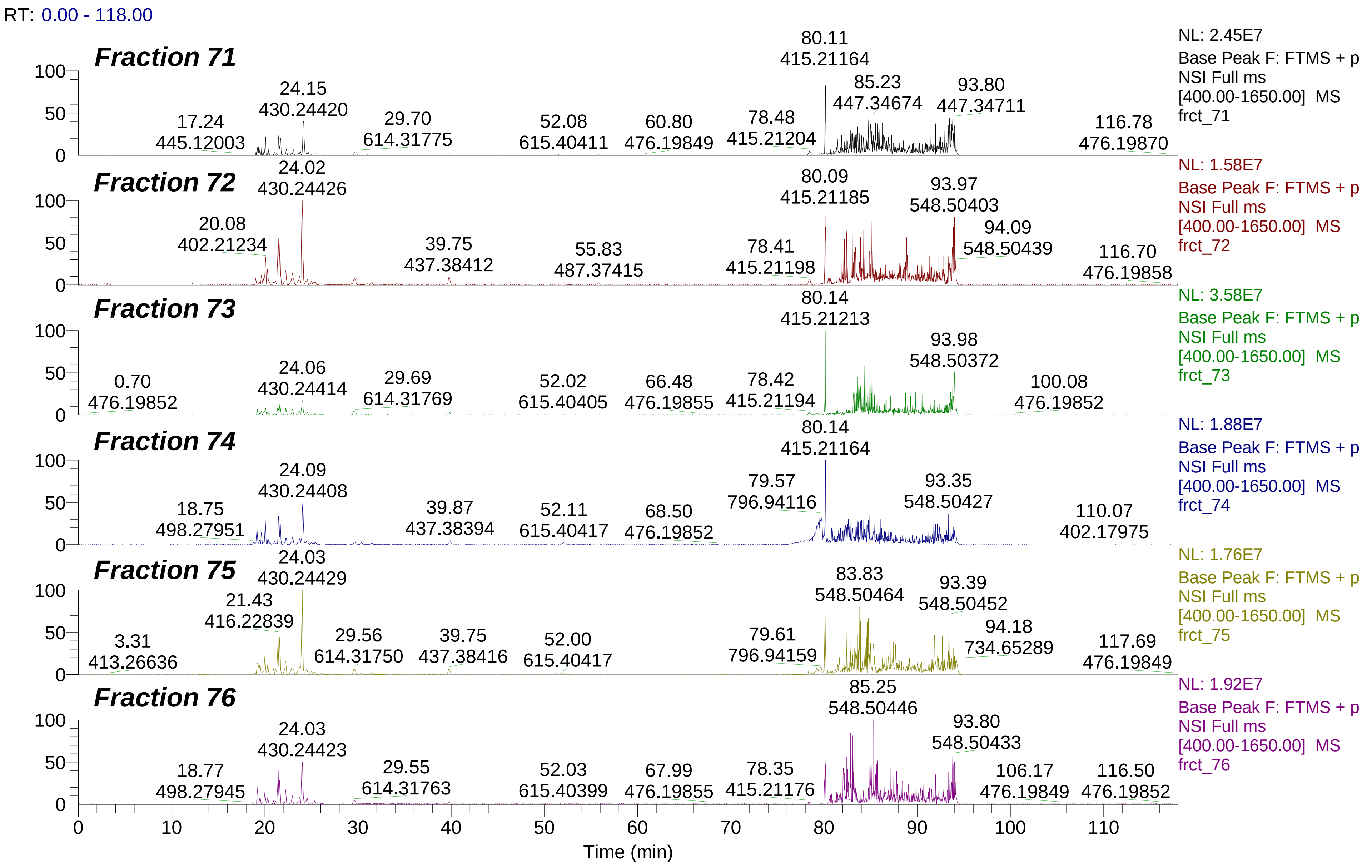

A. Full chromatograms (nano-LC) of isolated fractions from ApoA-I digested at arginine (R) residues (Part 2)

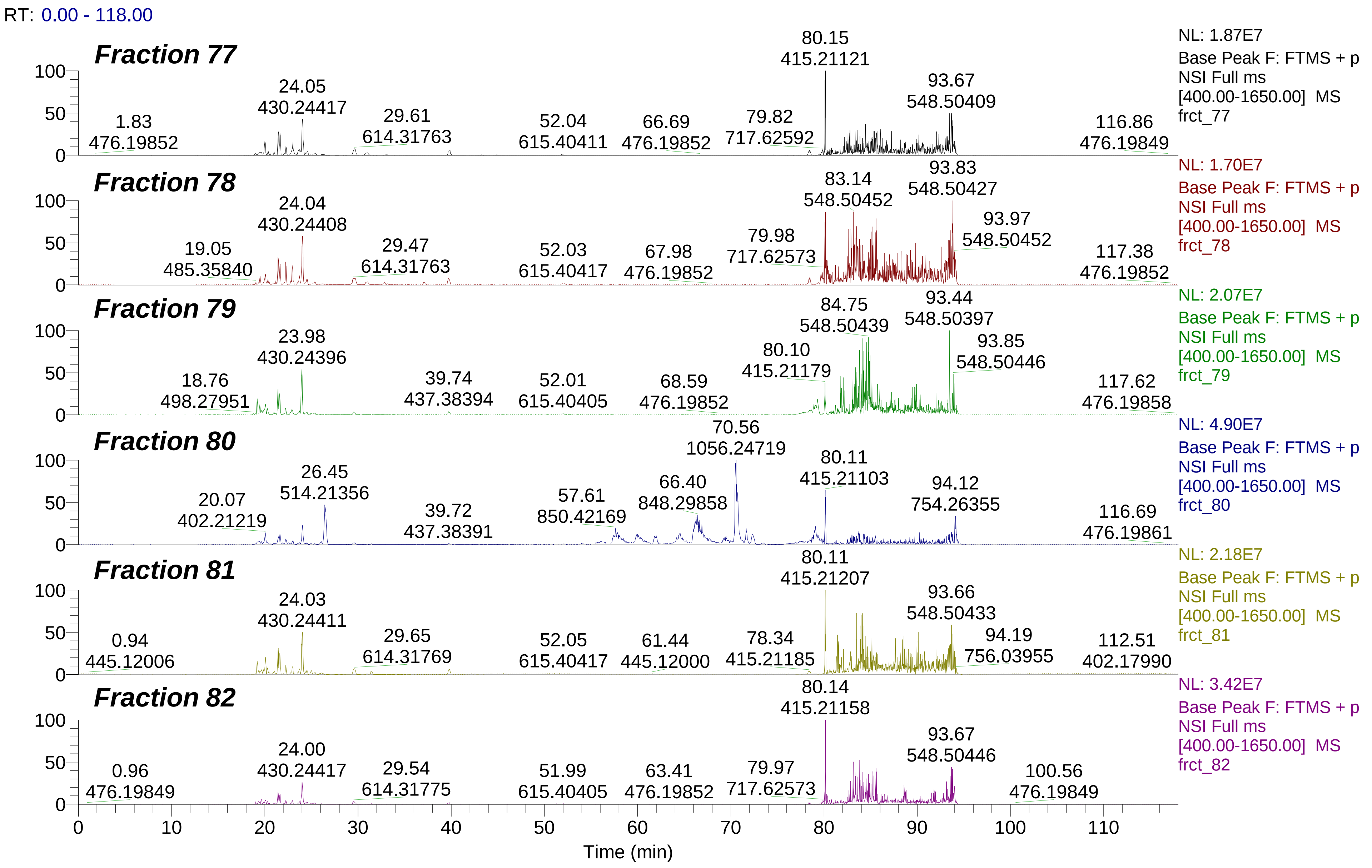

A. Full chromatograms (nano-LC) of isolated fractions from ApoA-I digested at arginine (R) residues (Part 3)

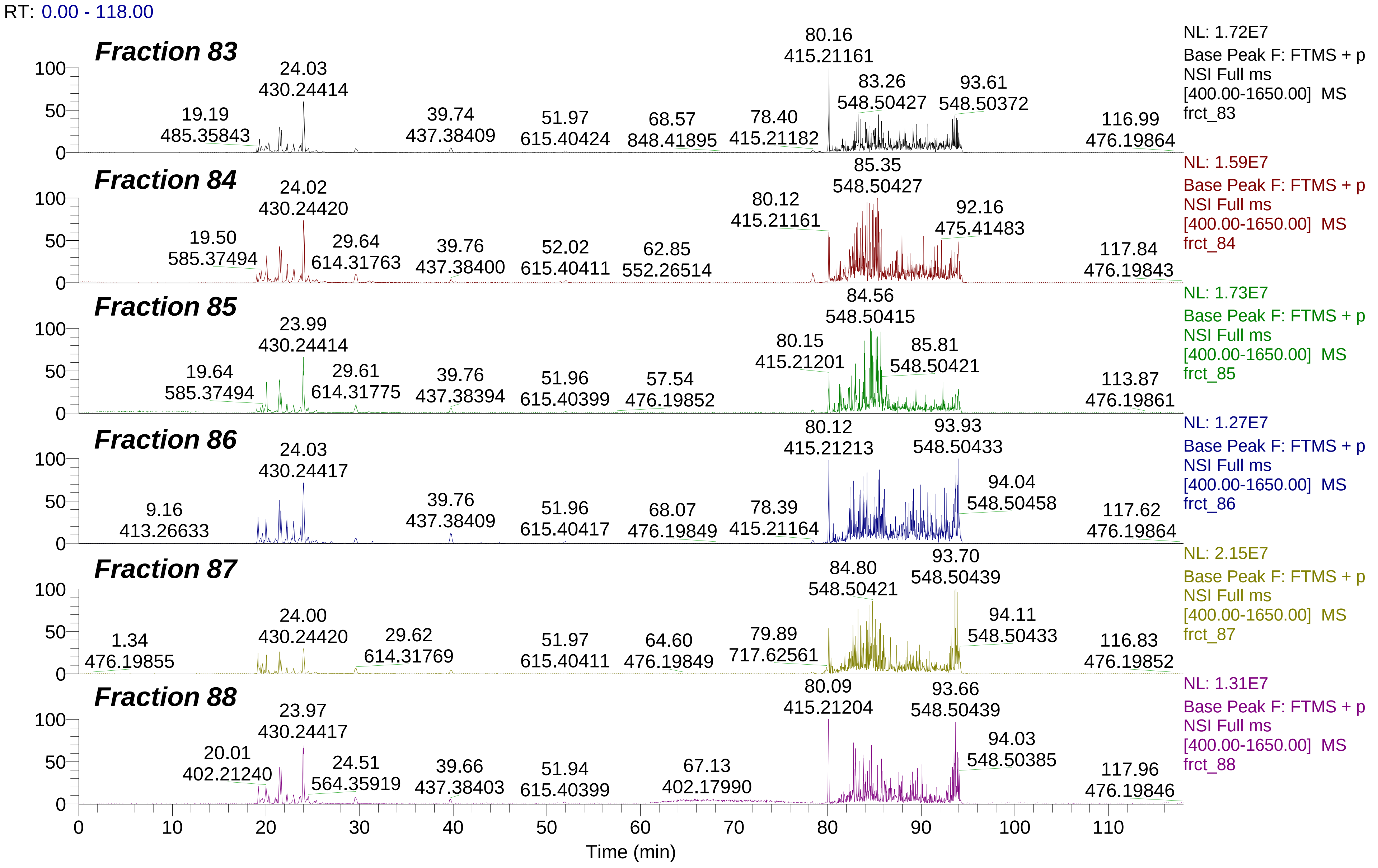

B. MS/MS - Fraction 74: 796.94 (4+); RT: 79.42 min (Parte 1)

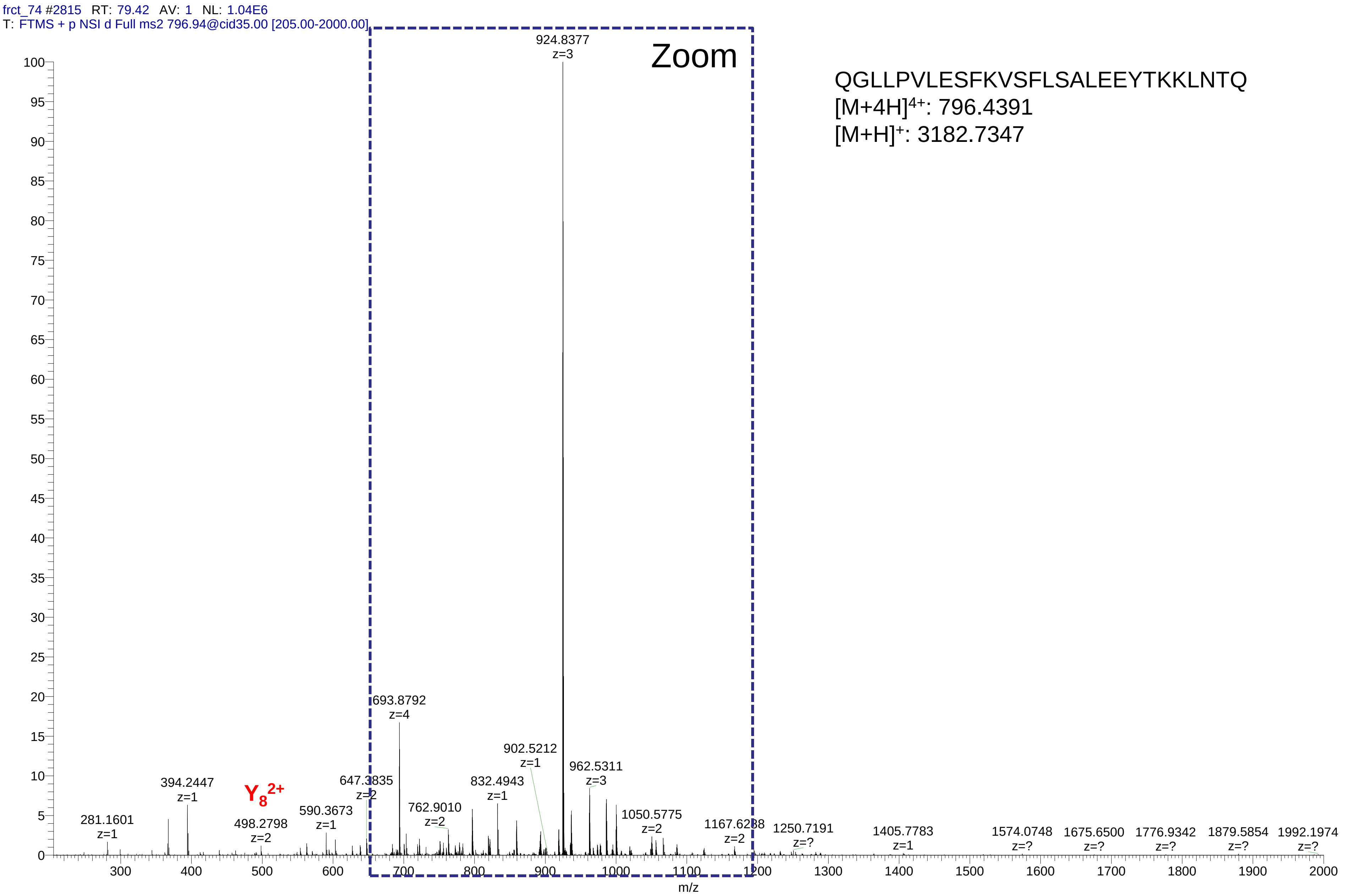

B. MS/MS - Fraction 74: 796.94 (4+); RT: 79.42 min (Parte 2 - ZOOM)

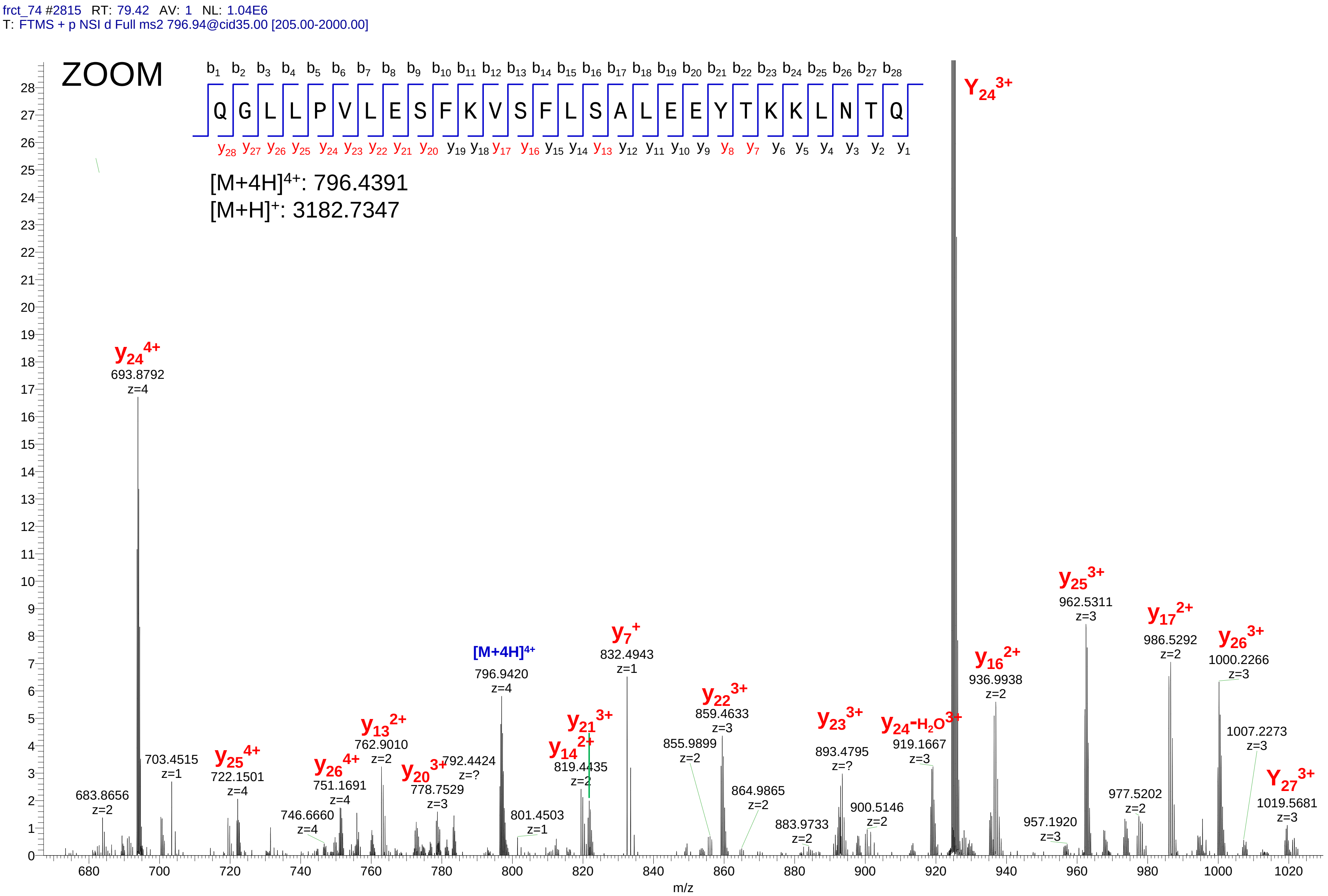

C. MS/MS - Fraction 74: m/z 1056.24 (3+); RT: 79.33 min

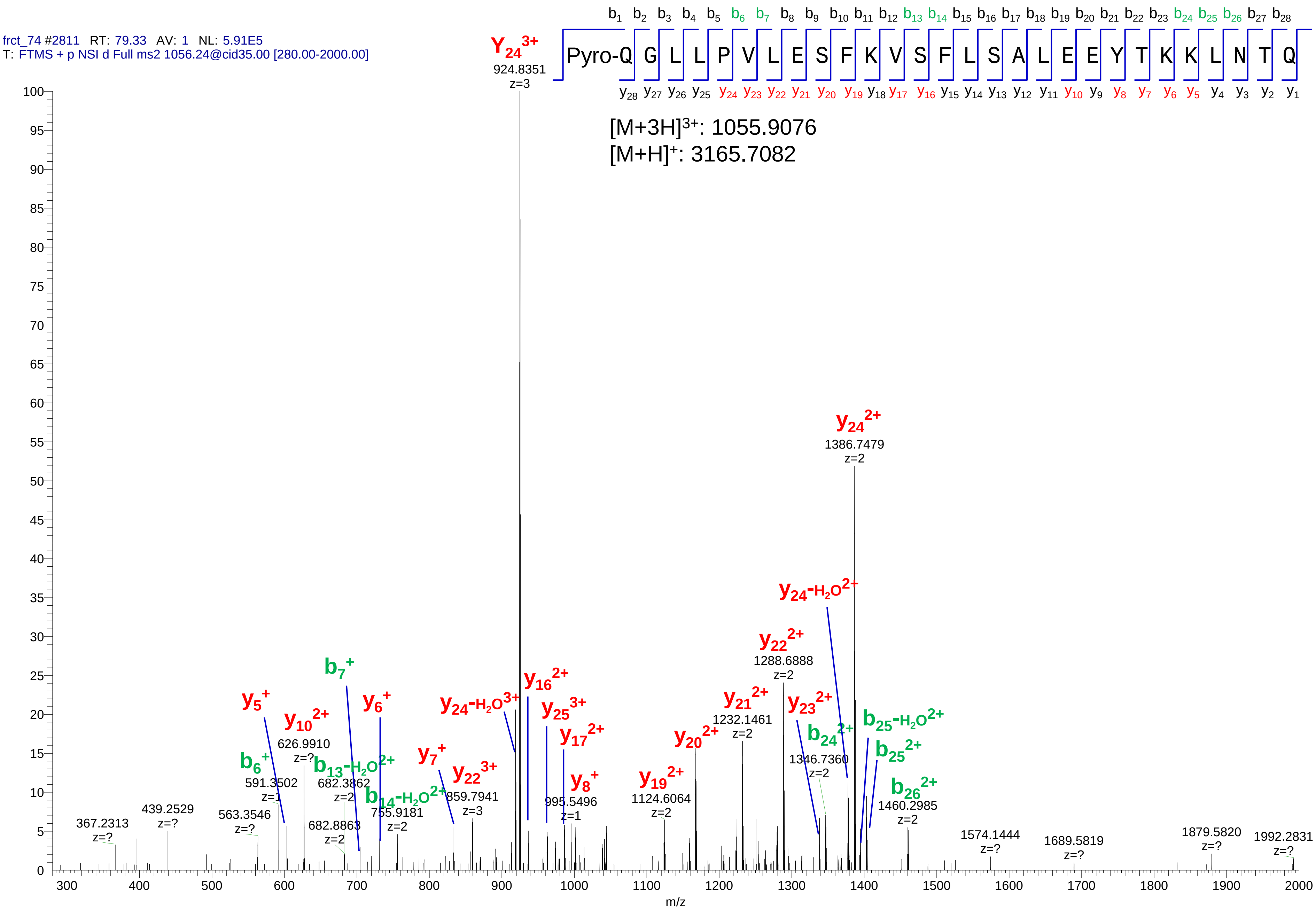

D. MS - Fraction 80 (RT: 61.70)

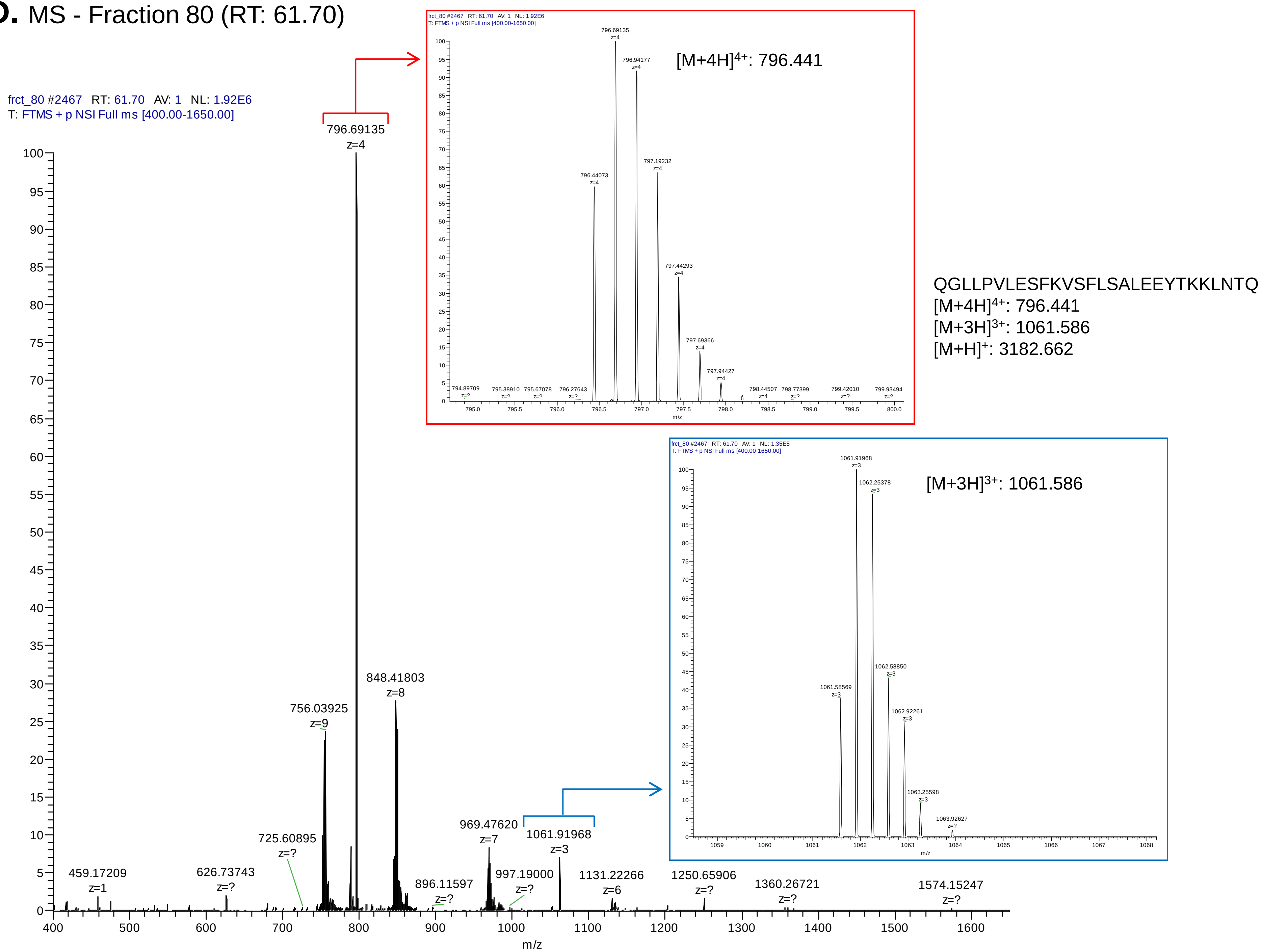

E. MS/MS - Fraction 80: m/z 796.69 (4+); RT: 61.76 min (Parte 1)

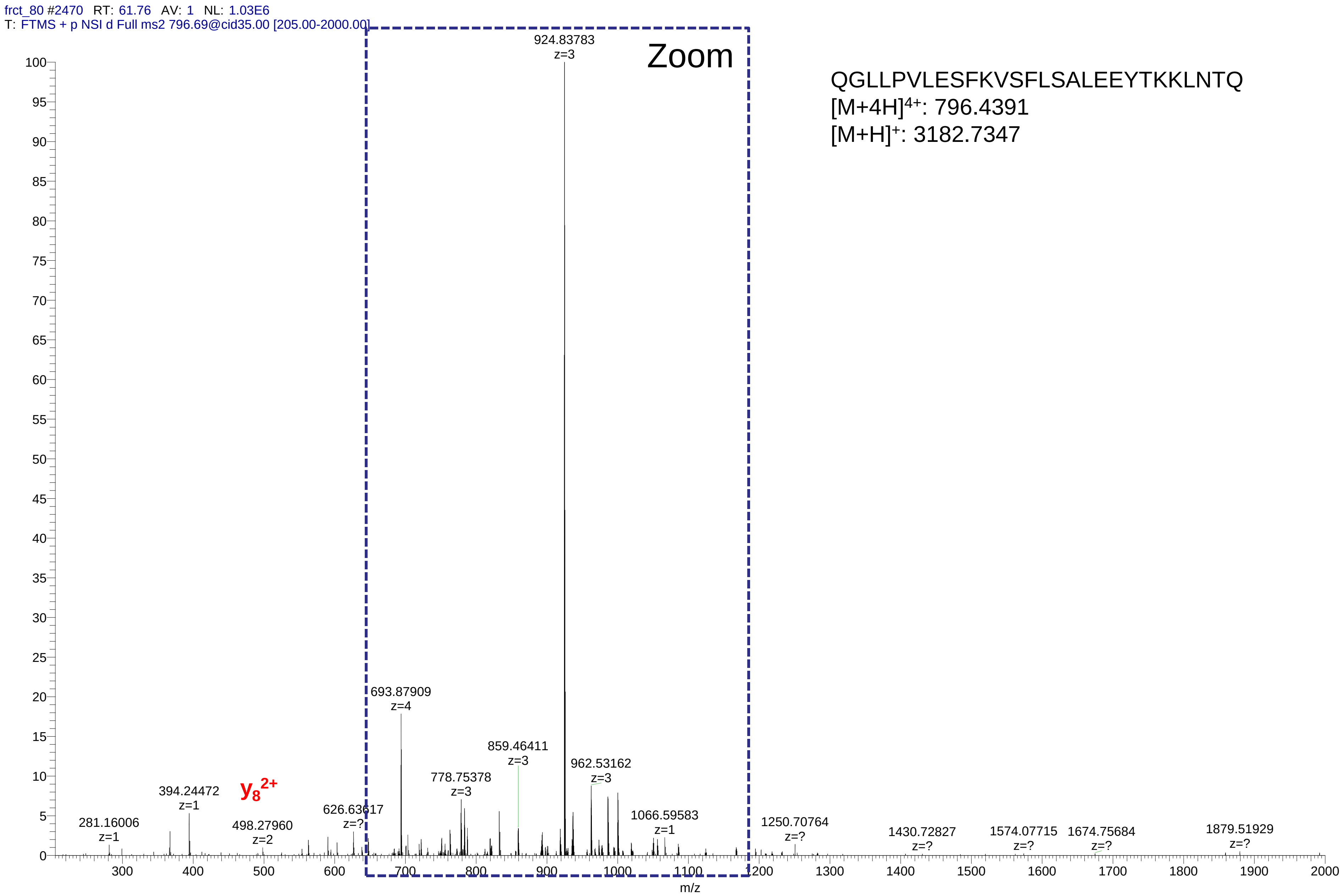

E. MS/MS - Fraction 80: m/z 796.69 (4+); RT: 61.76 min (Parte 2) (ZOOM)

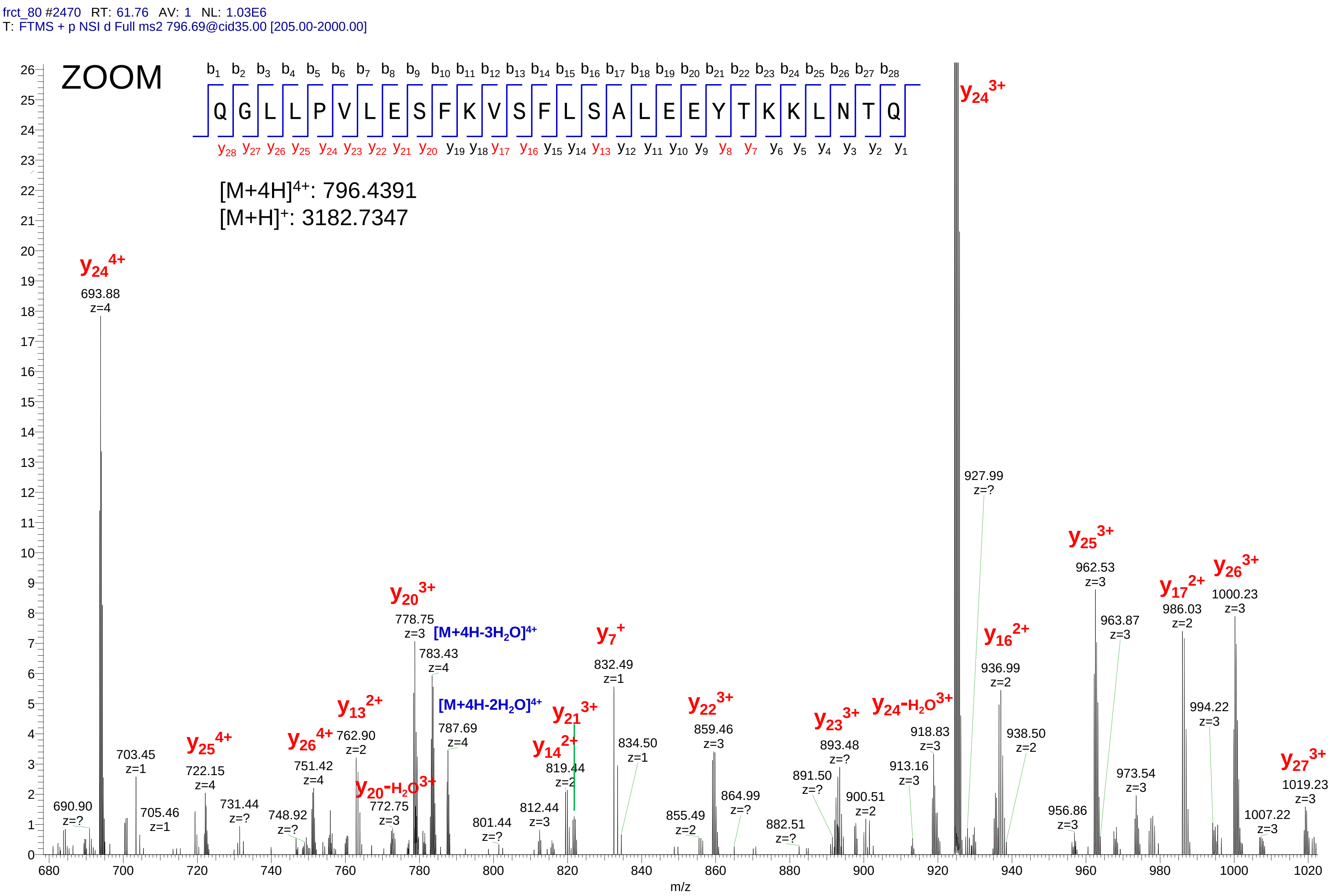

F. MS - Fraction 80 (RT: 70.65)

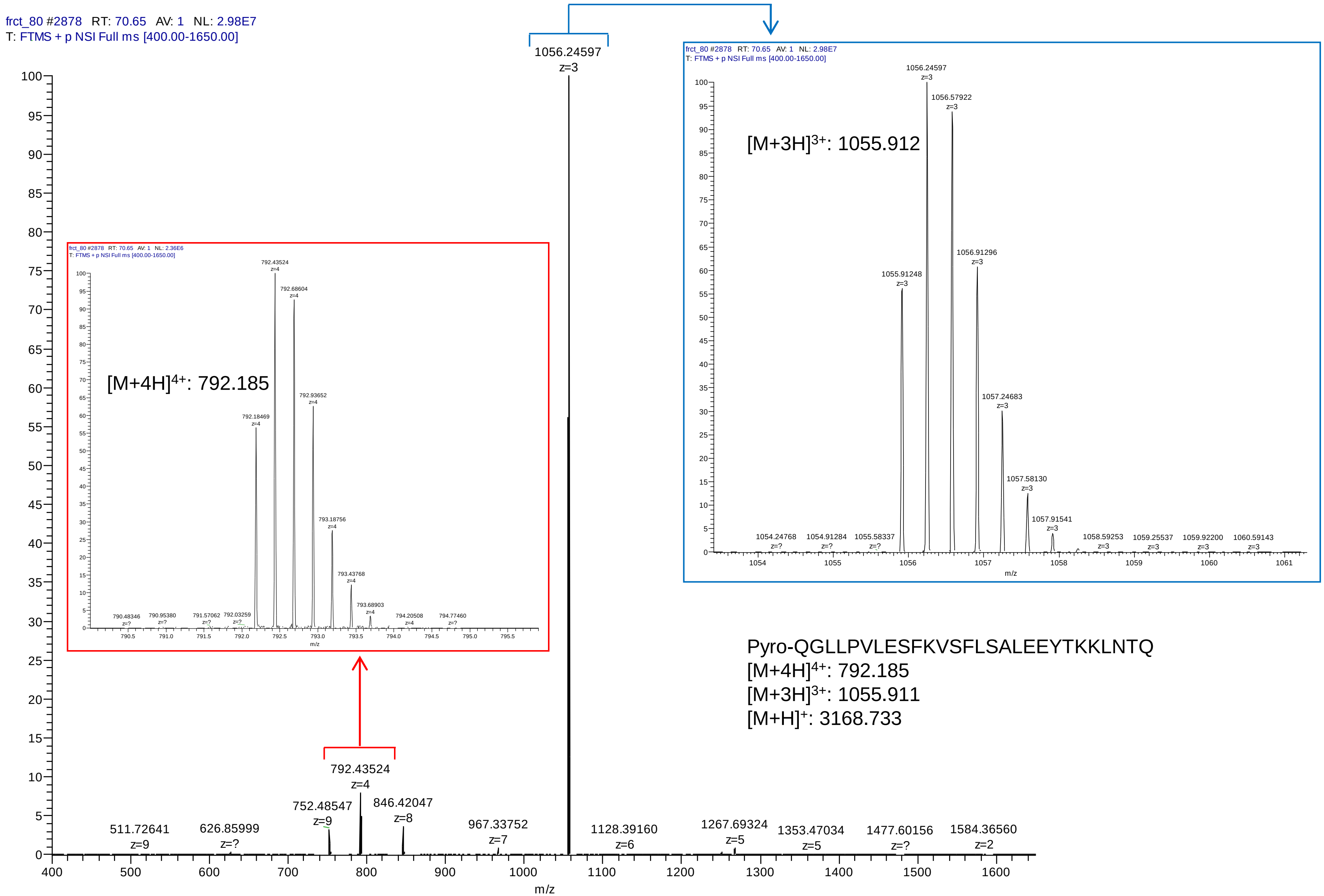

G. MS/MS - Fraction 80: m/z 1056.24 (3+); RT:70.57 min

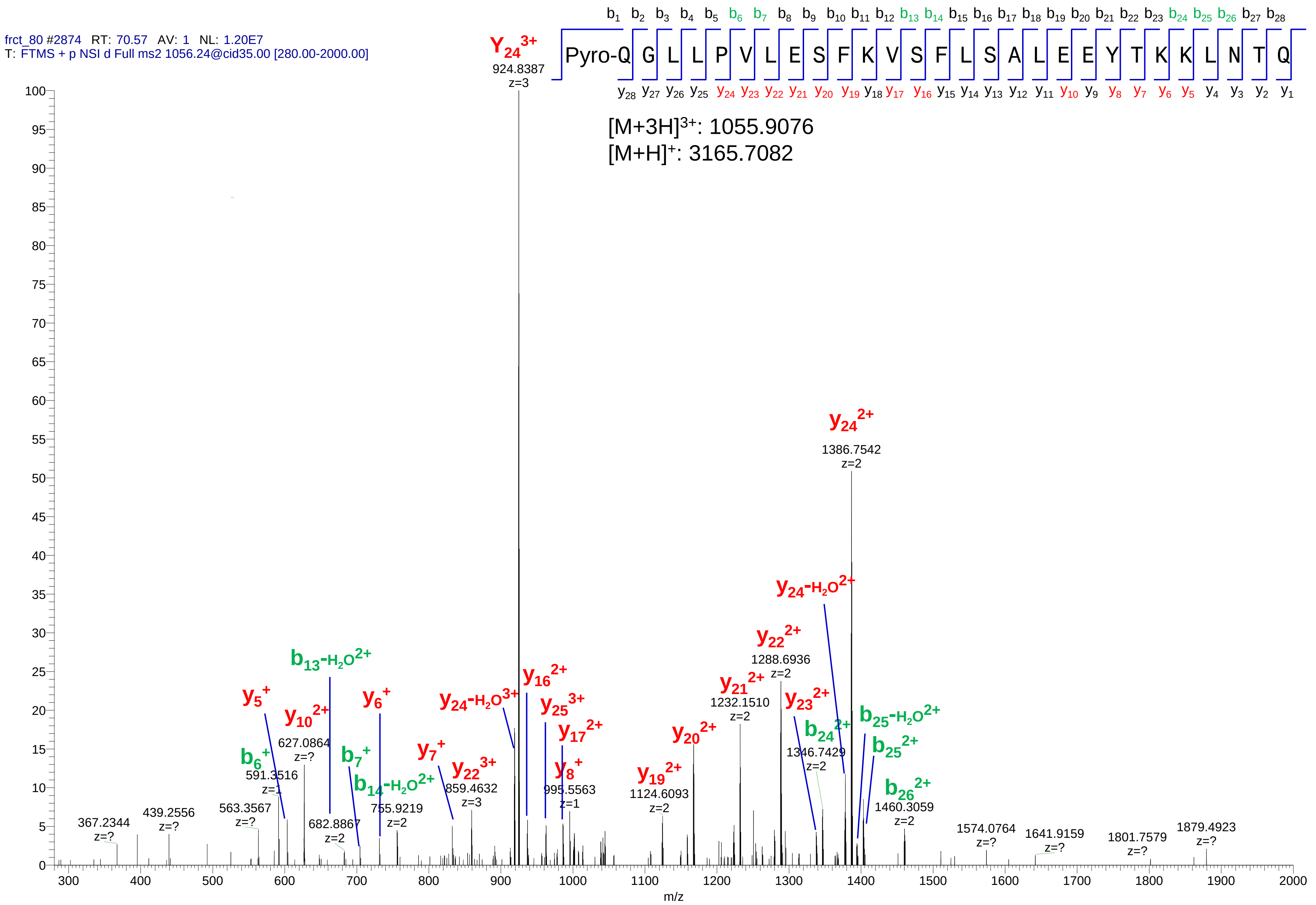

H. Extracted Ion Chromatogram of the peptide with m/z= 792.44 in of isolated fractions from ApoA-I digested at arginine (R) residues (Part 1)

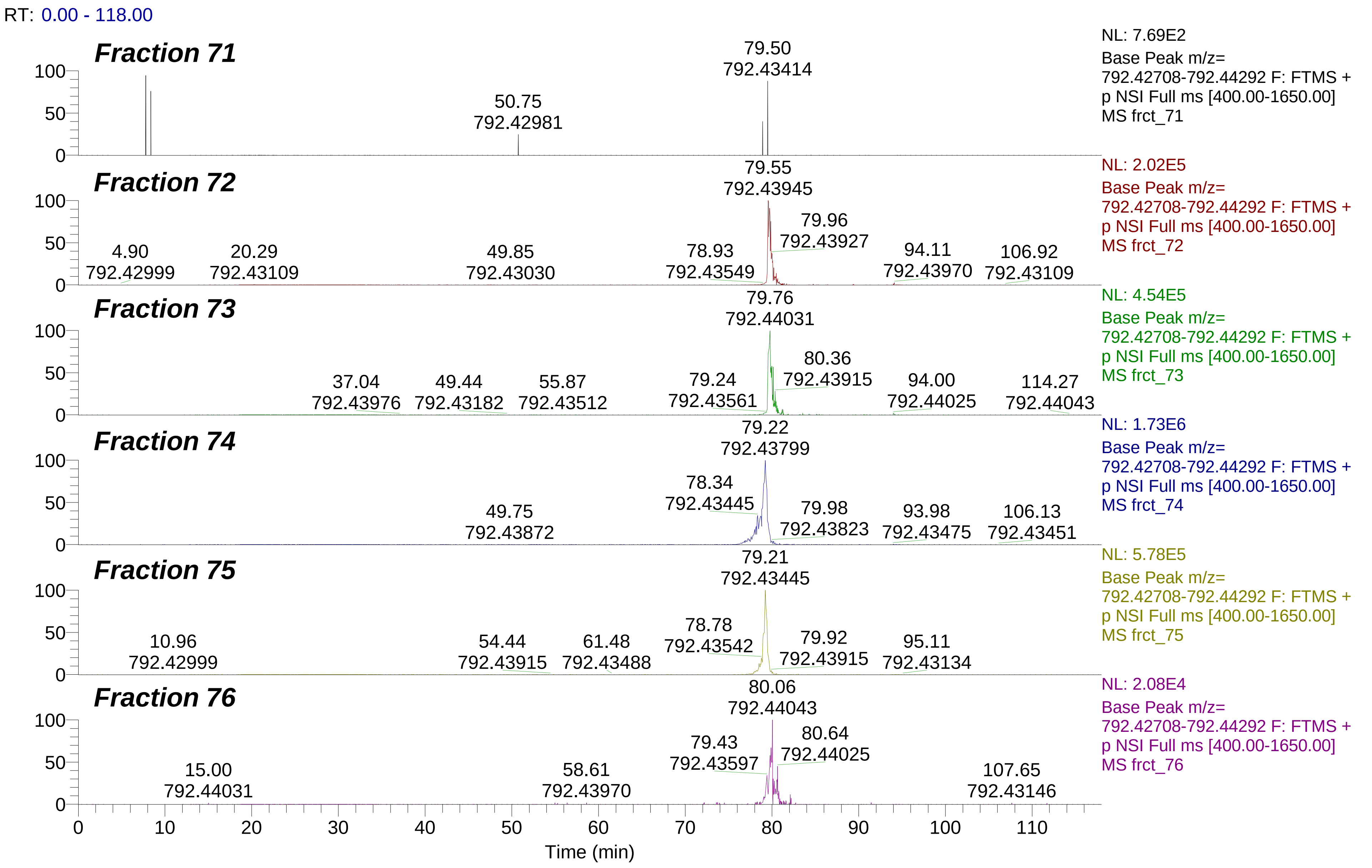

H. Extracted Ion Chromatogram of the peptide with m/z= 792.44 in of isolated fractions from ApoA-I digested at arginine (R) residues (Part 2)

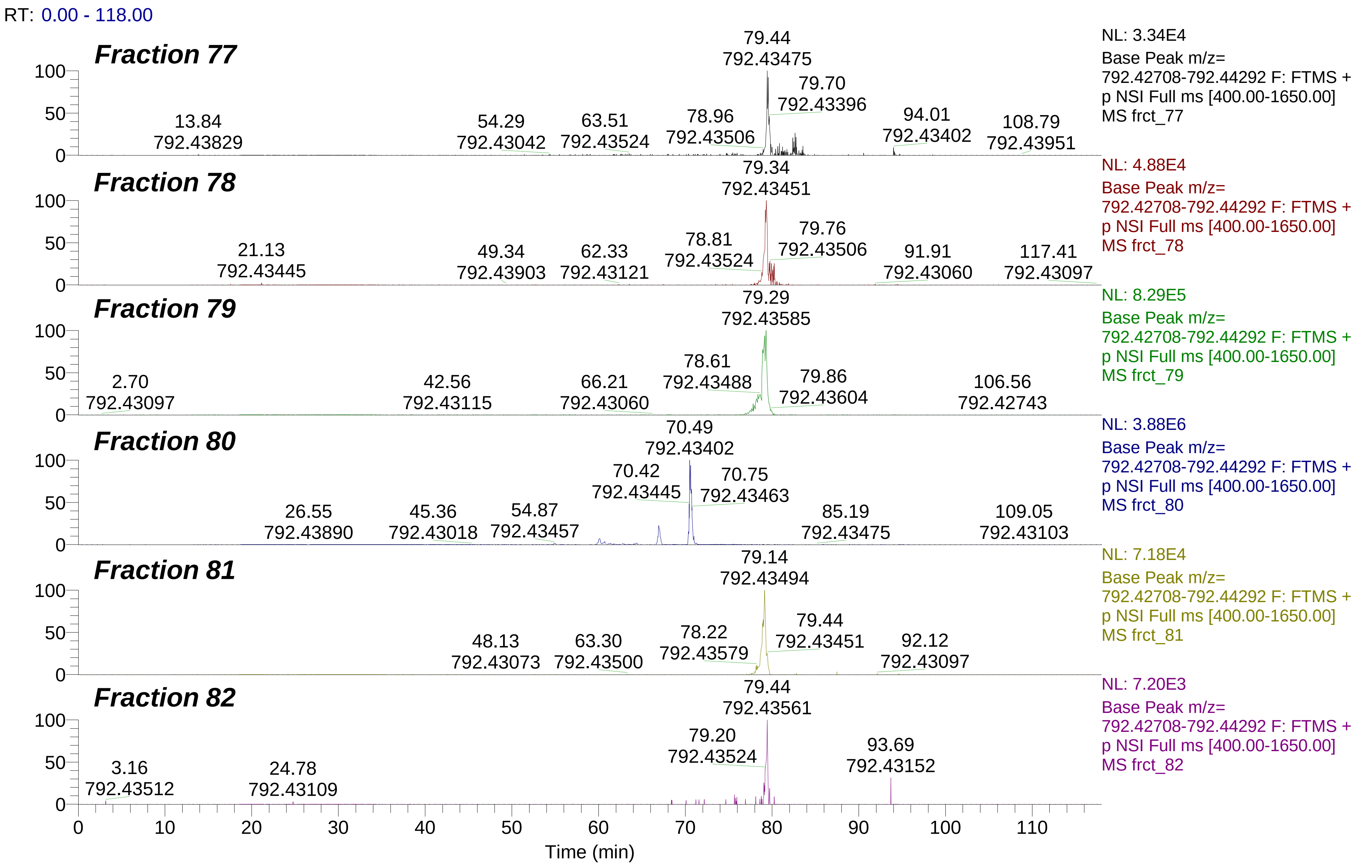

H. Extracted Ion Chromatogram of the peptide with m/z= 792.44 in of isolated fractions from ApoA-I digested at arginine (R) residues (Part 3)

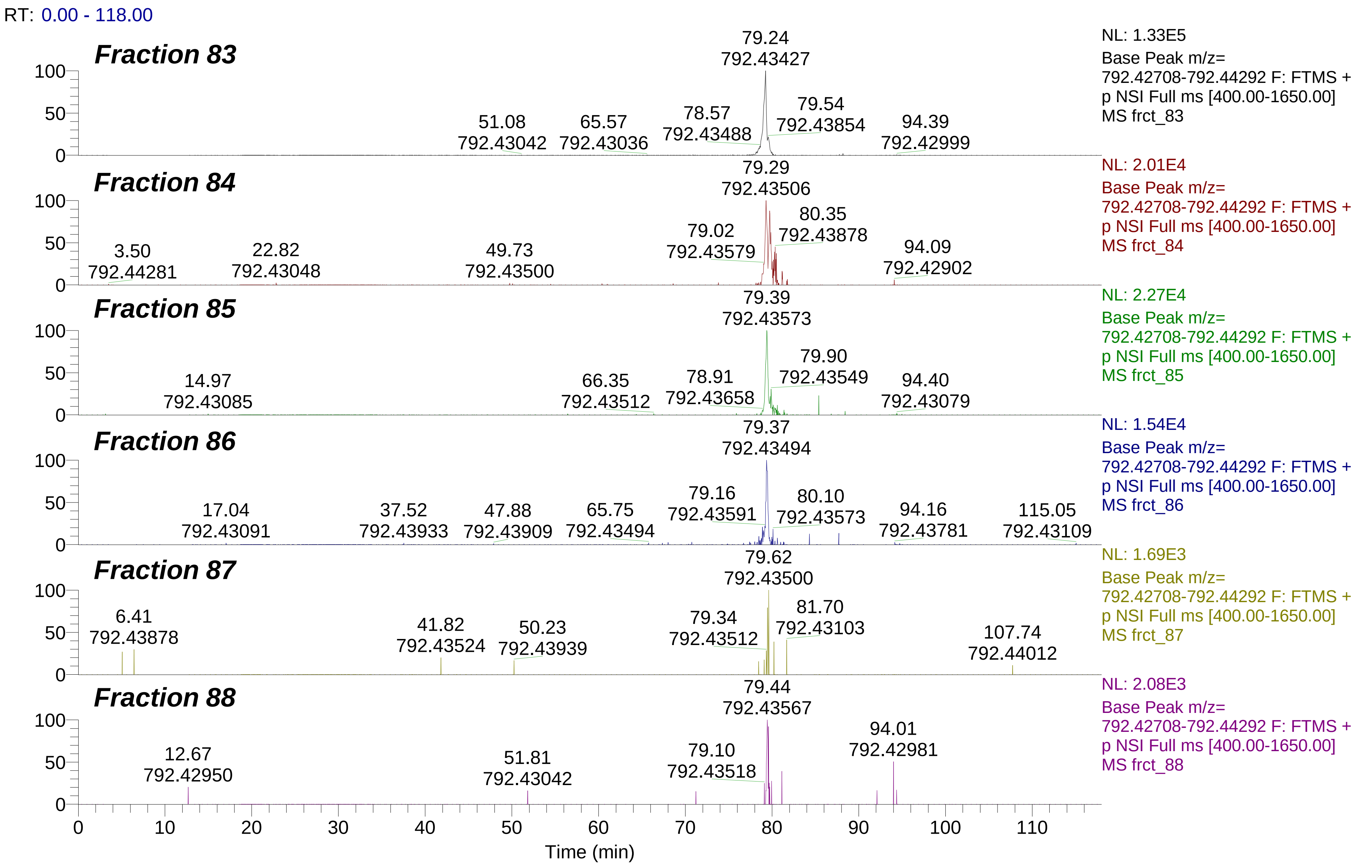

I. Extracted Ion Chromatogram of the peptide with m/z= 796.94 in of isolated fractions from ApoA-I digested at arginine (R) residues (Part 1)

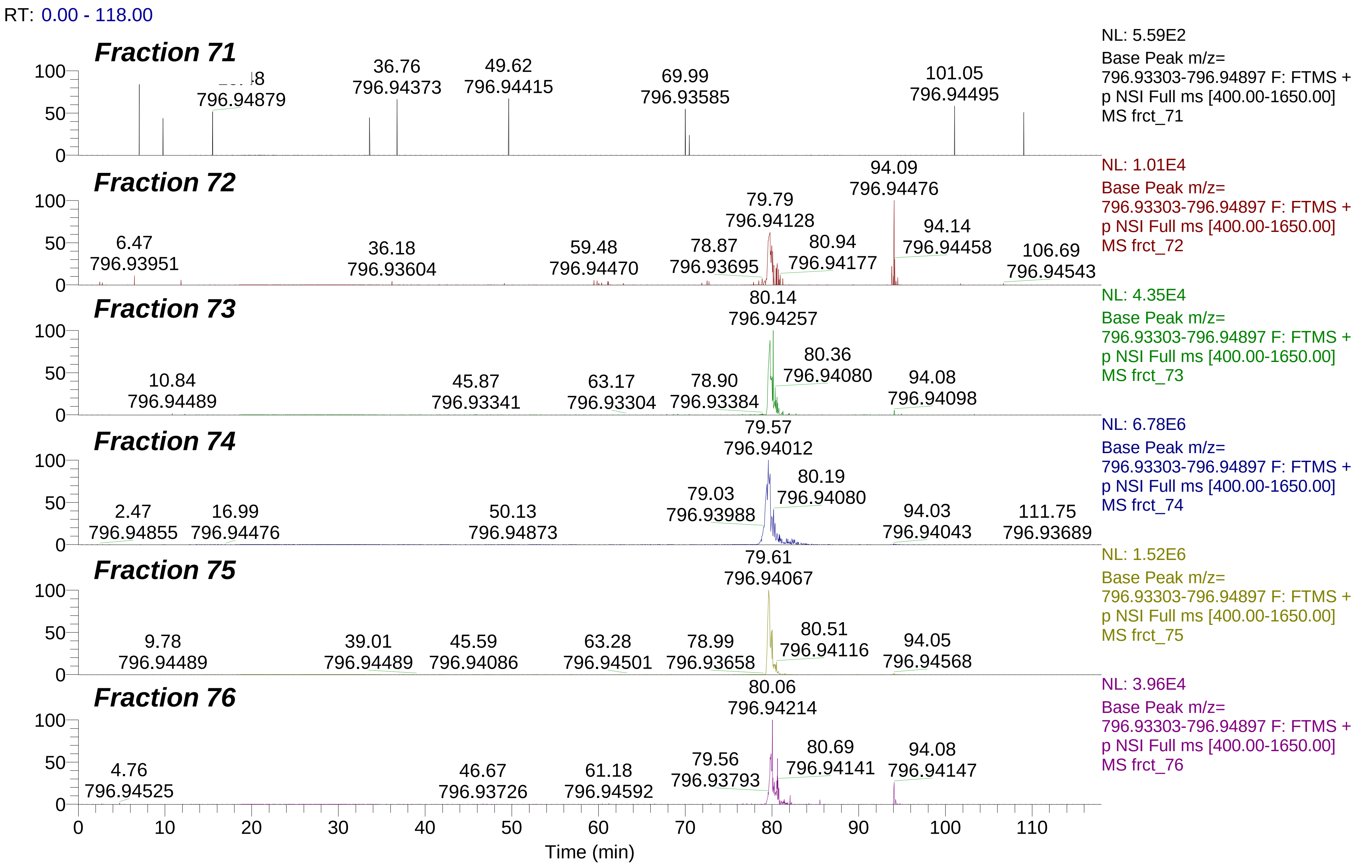

I. Extracted Ion Chromatogram of the peptide with m/z= 796.94 in of isolated fractions from ApoA-I digested at arginine (R) residues (Part 2)

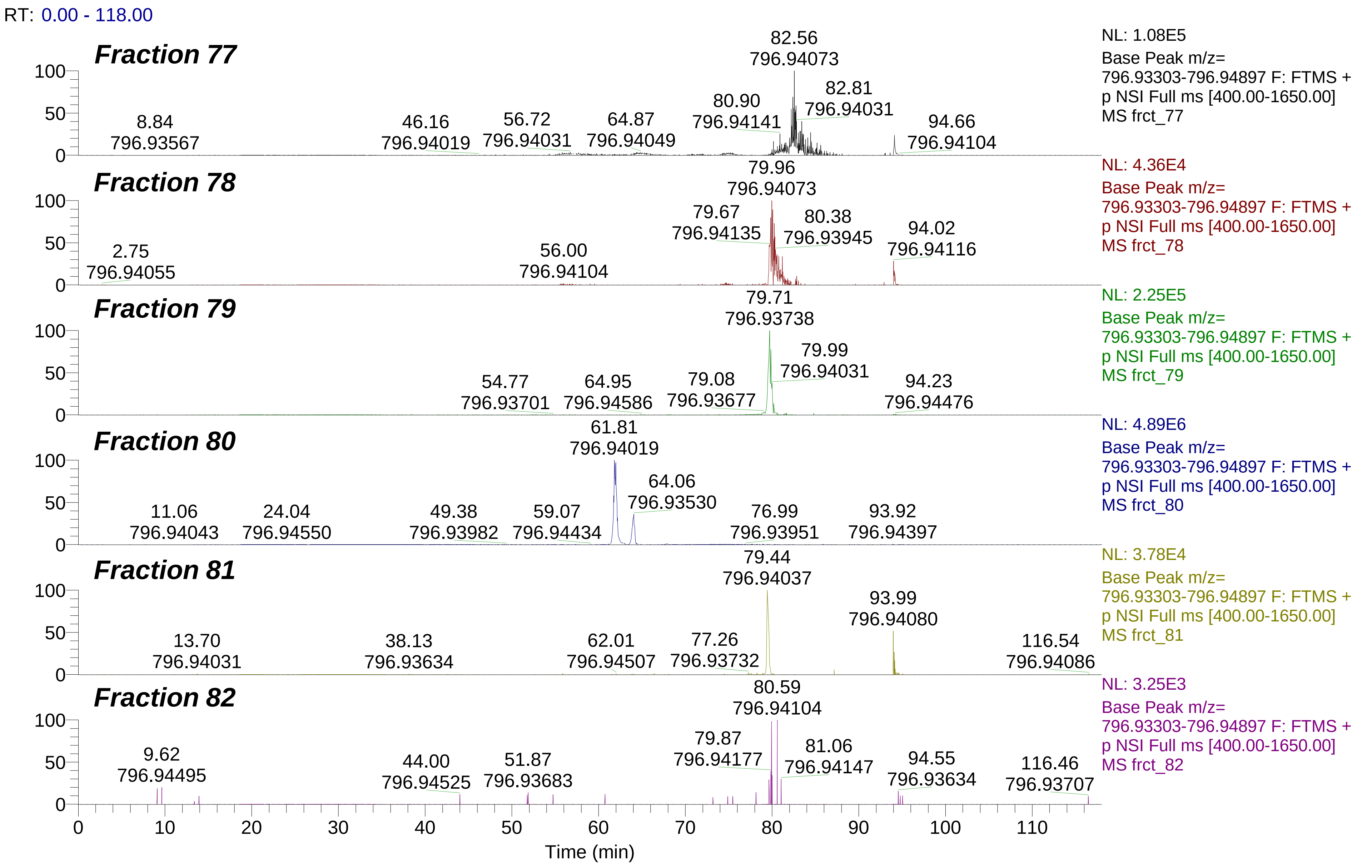

I. Extracted Ion Chromatogram of the peptide with m/z= 796.94 in of isolated fractions from ApoA-I digested at arginine (R) residues (Part 3)

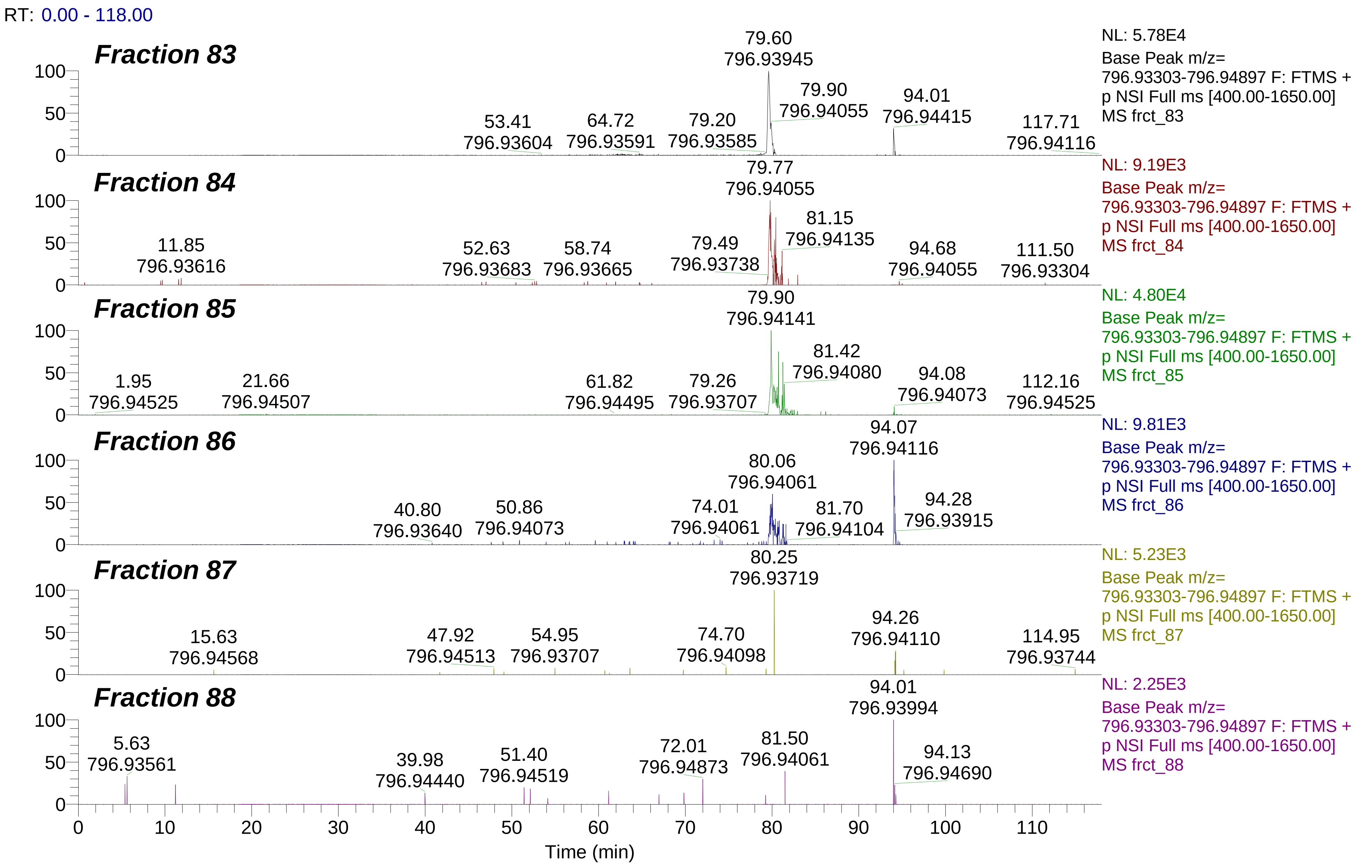

J. Extracted Ion Chromatogram of the peptide with m/z= 1056.24 in of isolated fractions from ApoA-I digested at arginine (R) residues (Part 1)

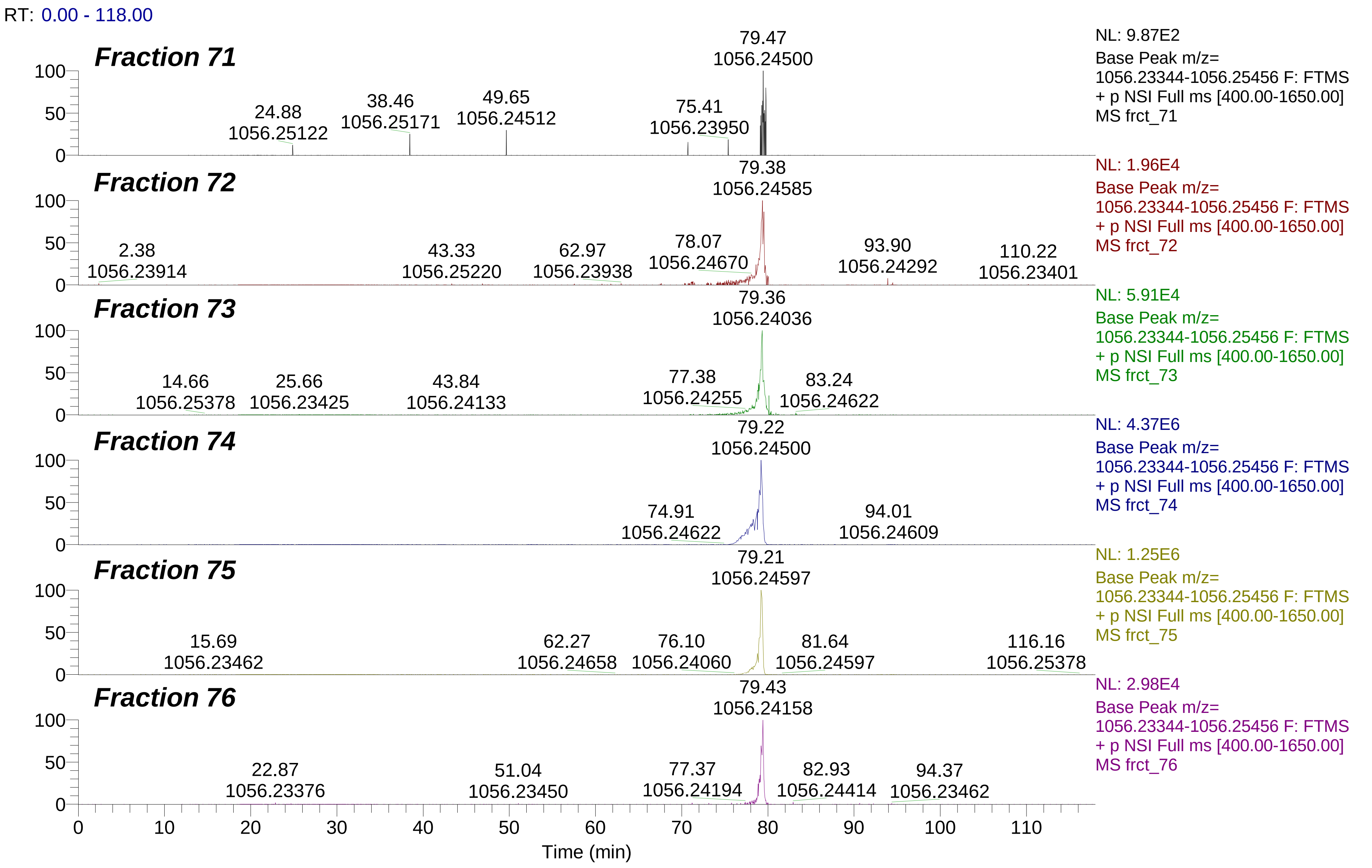

J. Extracted Ion Chromatogram of the peptide with m/z= 1056.24 in of isolated fractions from ApoA-I digested at arginine (R) residues (Part 2)

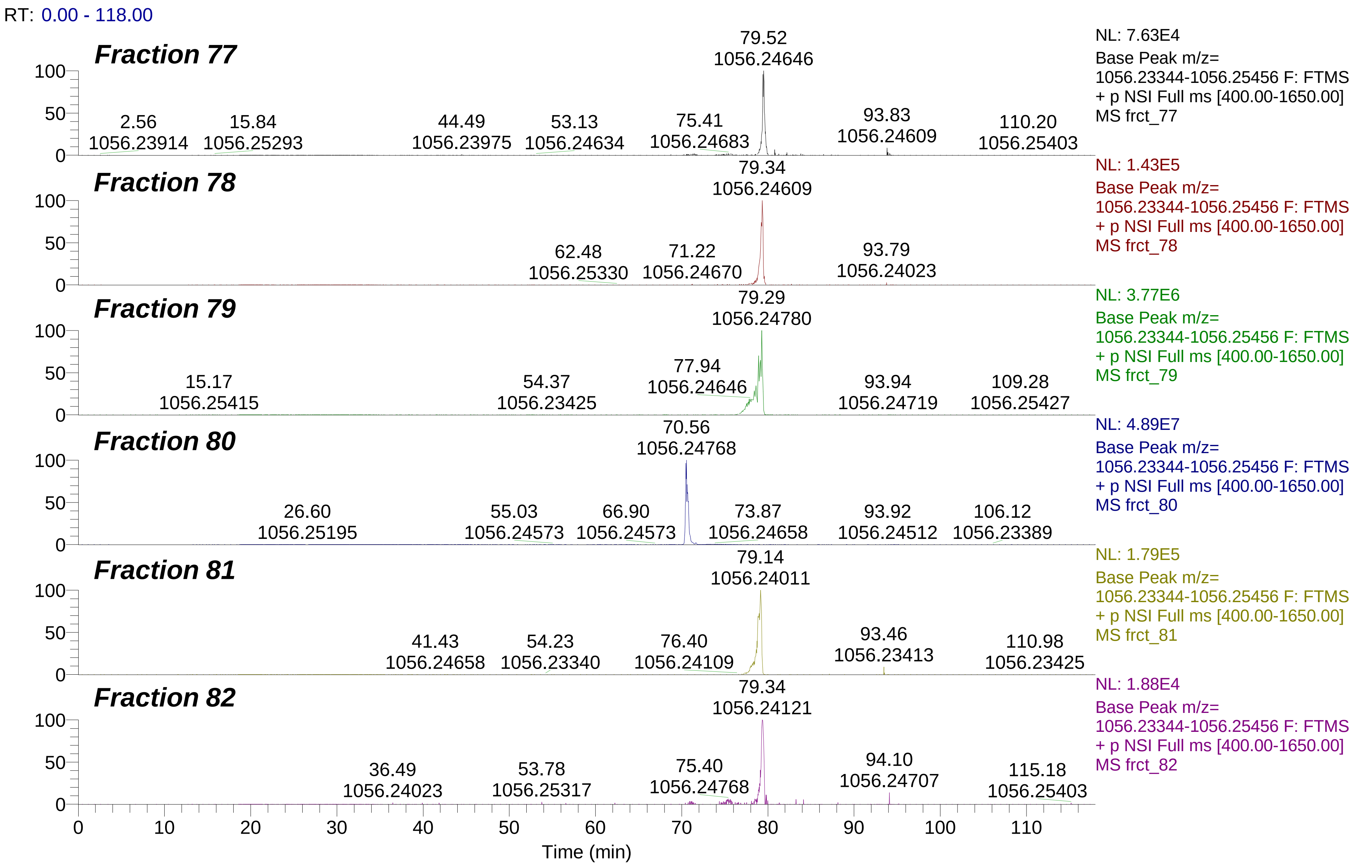

J. Extracted Ion Chromatogram of the peptide with m/z= 1056.24 in of isolated fractions from ApoA-I digested at arginine (R) residues (Part 3)

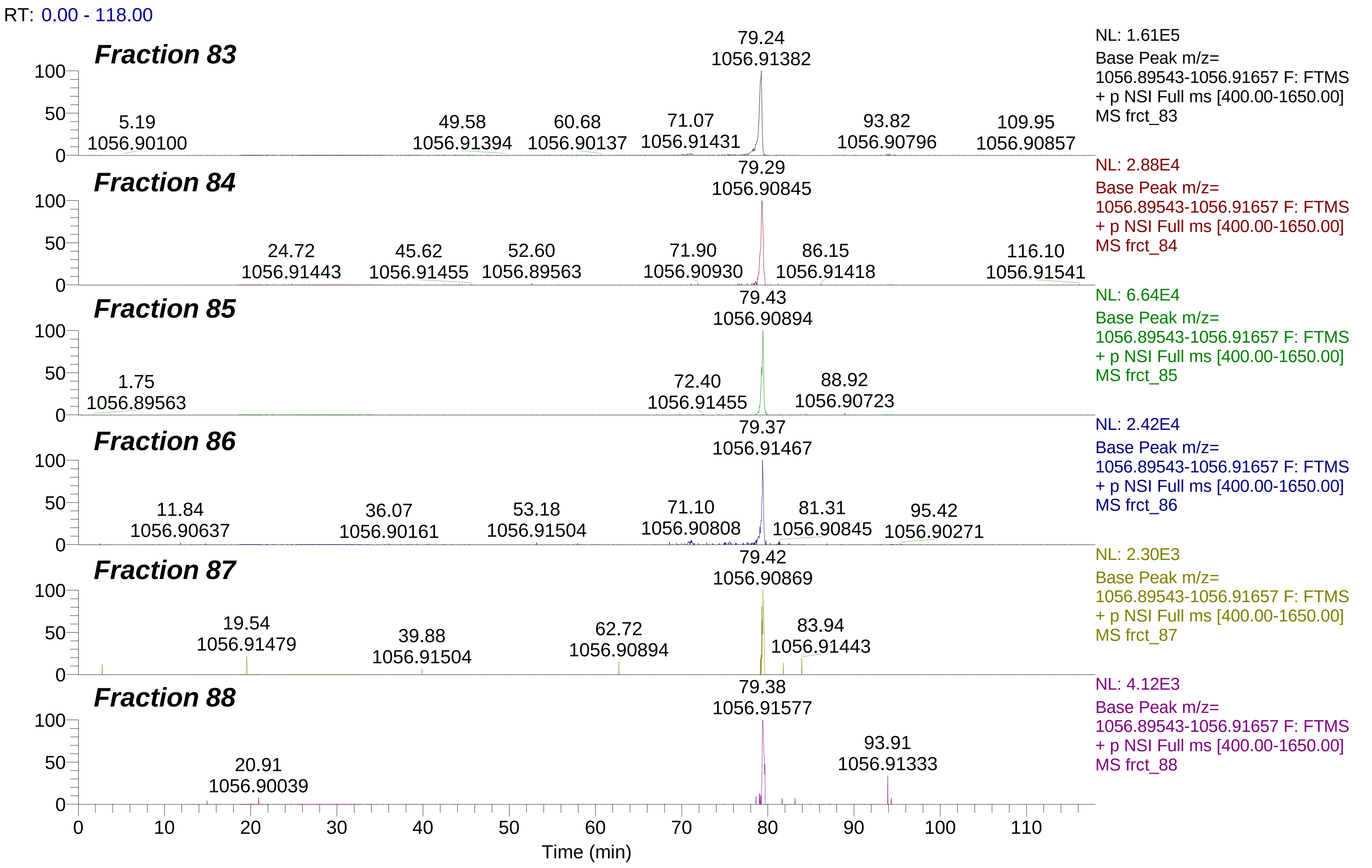

K. Extracted Ion Chromatogram of the peptide with m/z= 1061.91 in of isolated fractions from ApoA-I digested at arginine (R) residues (Part 1)

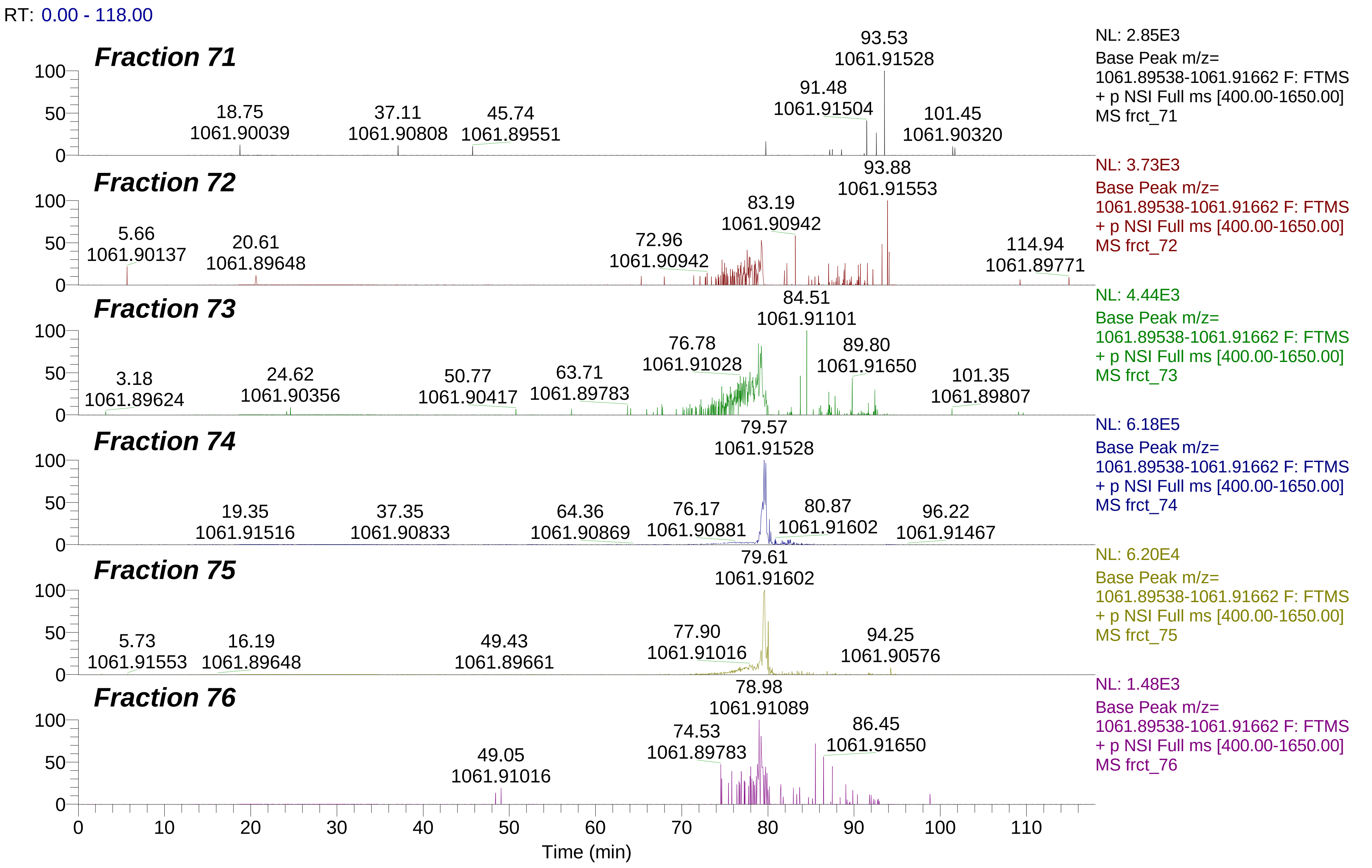

K. Extracted Ion Chromatogram of the peptide with m/z= 1061.91 in of isolated fractions from ApoA-I digested at arginine (R) residues (Part 2)

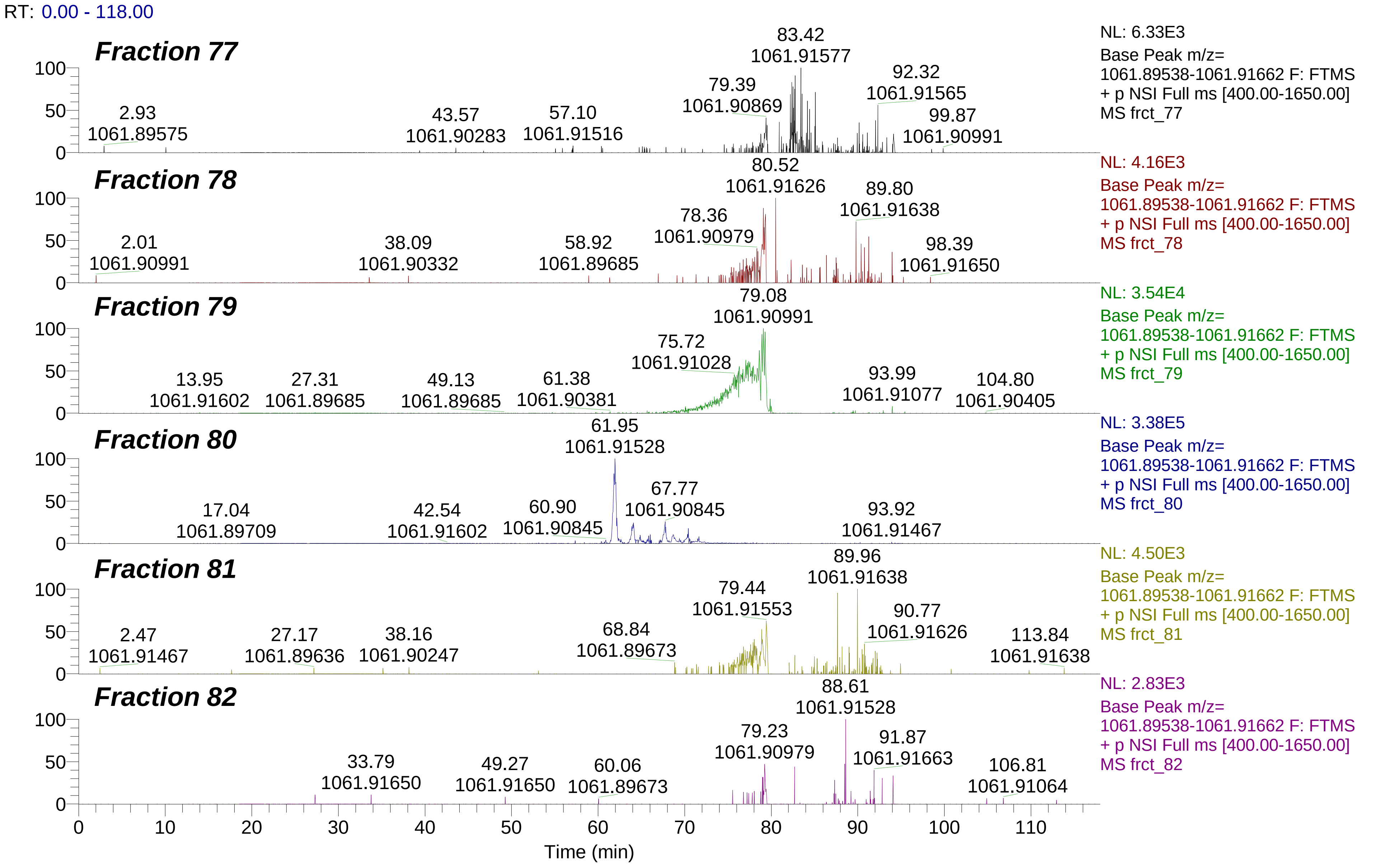

K. Extracted Ion Chromatogram of the peptide with m/z= 1061.91 in of isolated fractions from ApoA-I digested at arginine (R) residues (Part 3)

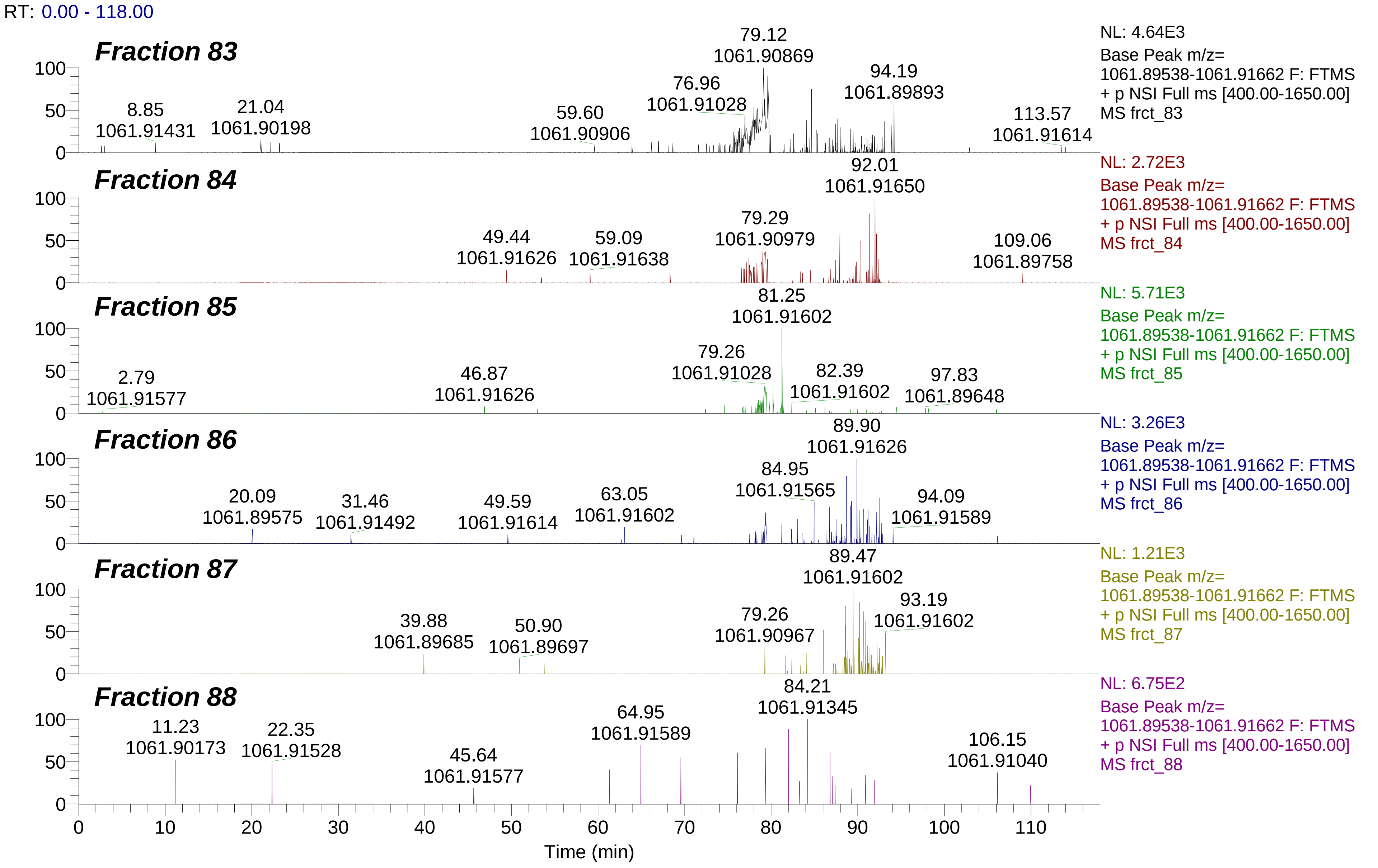

L. ApoA-I peptides identified in the fraction 80 using the Mascot Distiller tool.

Start	End	Observed	Mr(expt)	Mr(calc)	ppm	MC	Score	Expect	Peptide
240	267	1055.9100	3164.7082	3164.7009	2.3	0	119	1.3E-12	R.QGLLPVLESFKVSFLSALEEYTKKLNTQ.- + Gln->pyro-Glu (N-term Q)
240	267	796.4405	3181.7329	3181.7274	1.72	0	106	2.9E-11	R.QGLLPVLESFKVSFLSALEEYTKKLNTQ.-
240	267	1055.9123	3164.7151	3164.7009	4.48	0	102	6.8E-11	R.QGLLPVLESFKVSFLSALEEYTKKLNTQ.- + Gln->pyro-Glu (N-term Q)
240	267	1055.9101	3164.7085	3164.7009	2.39	0	100	1.1E-10	R.QGLLPVLESFKVSFLSALEEYTKKLNTQ.- + Gln->pyro-Glu (N-term Q)
240	267	1055.9099	3164.7079	3164.7009	2.2	0	80	1E-08	R.QGLLPVLESFKVSFLSALEEYTKKLNTQ.- + Gln->pyro-Glu (N-term Q)
240	267	796.4398	3181.7302	3181.7274	0.86	0	71	7.5E-08	R.QGLLPVLESFKVSFLSALEEYTKKLNTQ.-
240	267	1055.9098	3164.7076	3164.7009	2.11	0	66	2.7E-07	R.QGLLPVLESFKVSFLSALEEYTKKLNTQ.- + Gln->pyro-Glu (N-term Q)
240	267	1055.9099	3164.7079	3164.7009	2.2	0	66	2.3E-07	R.QGLLPVLESFKVSFLSALEEYTKKLNTQ.- + Gln->pyro-Glu (N-term Q)
86	140	1127.5561	6759.2929	6759.2788	2.09	1	65	4.2E-07	R.EQLGPVTQEFWDNLEKETEGLRQEMSKDLEEVKAKVQPYLDDFQKKWQEEMELYR.Q
240	267	1055.9098	3164.7076	3164.7009	2.11	0	64	4E-07	R.QGLLPVLESFKVSFLSALEEYTKKLNTQ.- + Gln->pyro-Glu (N-term Q)
86	140	1130.2221	6775.2889	6775.2737	2.24	1	59	1.8E-06	R.EQLGPVTQEFWDNLEKETEGLRQEMSKDLEEVKAKVQPYLDDFQKKWQEEMELYR.Q + Oxidation (M)
86	140	1130.2218	6775.2871	6775.2737	1.98	1	58	2.5E-06	R.EQLGPVTQEFWDNLEKETEGLRQEMSKDLEEVKAKVQPYLDDFQKKWQEEMELYR.Q + Oxidation (M)
86	140	1130.2242	6775.3015	6775.2737	4.1	1	57	3E-06	R.EQLGPVTQEFWDNLEKETEGLRQEMSKDLEEVKAKVQPYLDDFQKKWQEEMELYR.Q + Oxidation (M)
86	140	1132.8908	6791.3011	6791.2687	4.78	1	55	5.1E-06	R.EQLGPVTQEFWDNLEKETEGLRQEMSKDLEEVKAKVQPYLDDFQKKWQEEMELYR.Q + 2 Oxidation (M)
86	140	1130.2218	6775.2871	6775.2737	1.98	1	53	8.1E-06	R.EQLGPVTQEFWDNLEKETEGLRQEMSKDLEEVKAKVQPYLDDFQKKWQEEMELYR.Q + Oxidation (M)
240	267	1061.5859	3181.7359	3181.7274	2.65	0	53	5.6E-06	R.QGLLPVLESFKVSFLSALEEYTKKLNTQ.
86	140	1130.2217	6775.2865	6775.2737	1.89	1	52	8.6E-06	R.EQLGPVTQEFWDNLEKETEGLRQEMSKDLEEVKAKVQPYLDDFQKKWQEEMELYR.Q + Oxidation (M)
240	267	792.1841	3164.7074	3164.7009	2.06	0	49	1.3E-05	R.QGLLPVLESFKVSFLSALEEYTKKLNTQ.- + Gln->pyro-Glu (N-term Q)
86	140	1130.2217	6775.2865	6775.2737	1.89	1	44	6.3E-05	R.EQLGPVTQEFWDNLEKETEGLRQEMSKDLEEVKAKVQPYLDDFQKKWQEEMELYR.Q + Oxidation (M)
35	51	626.6829	1877.0268	1877.0258	0.49	0	40	0.00015	R.VKDLATVYYDVLKDSGR.D
86	140	1356.0686	6775.3066	6775.2737	4.85	1	40	0.00015	R.EQLGPVTQEFWDNLEKETEGLRQEMSKDLEEVKAKVQPYLDDFQKKWQEEMELYR.Q + Oxidation (M)
86	140	1127.5562	6759.2935	6759.2788	2.18	1	38	0.00021	R.EQLGPVTQEFWDNLEKETEGLRQEMSKDLEEVKAKVQPYLDDFQKKWQEEMELYR.Q
86	140	1127.5558	6759.2911	6759.2788	1.82	1	37	0.00023	R.EQLGPVTQEFWDNLEKETEGLRQEMSKDLEEVKAKVQPYLDDFQKKWQEEMELYR.Q
86	140	971.1903	6791.2812	6791.2687	1.85	1	36	0.00034	R.EQLGPVTQEFWDNLEKETEGLRQEMSKDLEEVKAKVQPYLDDFQKKWQEEMELYR.Q + 2 Oxidation (M)
240	267	1055.9098	3164.7076	3164.7009	2.11	0	36	0.00028	R.QGLLPVLESFKVSFLSALEEYTKKLNTQ.- + Gln->pyro-Glu (N-term Q)
240	267	1055.9099	3164.7079	3164.7009	2.2	0	32	0.00065	R.QGLLPVLESFKVSFLSALEEYTKKLNTQ.- + Gln->pyro-Glu (N-term Q)
86	140	1356.0648	6775.2876	6775.2737	2.05	1	30	0.0014	R.EQLGPVTQEFWDNLEKETEGLRQEMSKDLEEVKAKVQPYLDDFQKKWQEEMELYR.Q + Oxidation (M)
86	140	1127.5559	6759.2917	6759.2788	1.91	1	28	0.0018	R.EQLGPVTQEFWDNLEKETEGLRQEMSKDLEEVKAKVQPYLDDFQKKWQEEMELYR.Q
240	267	792.1839	3164.7066	3164.7009	1.81	0	26	0.0027	R.QGLLPVLESFKVSFLSALEEYTKKLNTQ.- + Gln->pyro-Glu (N-term Q)
86	140	1130.2208	6775.2811	6775.2737	1.09	1	23	0.0072	R.EQLGPVTQEFWDNLEKETEGLRQEMSKDLEEVKAKVQPYLDDFQKKWQEEMELYR.Q + Oxidation (M)
86	140	1356.0638	6775.2826	6775.2737	1.31	1	23	0.0084	R.EQLGPVTQEFWDNLEKETEGLRQEMSKDLEEVKAKVQPYLDDFQKKWQEEMELYR.Q + Oxidation (M)
86	140	1127.5556	6759.2899	6759.2788	1.65	1	22	0.0083	R.EQLGPVTQEFWDNLEKETEGLRQEMSKDLEEVKAKVQPYLDDFQKKWQEEMELYR.Q
86	140	1130.2217	6775.2865	6775.2737	1.89	1	22	0.01	R.EQLGPVTQEFWDNLEKETEGLRQEMSKDLEEVKAKVQPYLDDFQKKWQEEMELYR.Q + Oxidation (M)
86	140	971.1909	6791.2854	6791.2687	2.47	1	22	0.0073	R.EQLGPVTQEFWDNLEKETEGLRQEMSKDLEEVKAKVQPYLDDFQKKWQEEMELYR.Q + 2 Oxidation (M)
86	140	1132.8877	6791.2825	6791.2687	2.04	1	21	0.009	R.EQLGPVTQEFWDNLEKETEGLRQEMSKDLEEVKAKVQPYLDDFQKKWQEEMELYR.Q + 2 Oxidation (M)
240	267	792.1843	3164.7080	3164.7009	2.25	0	21	0.0072	R.QGLLPVLESFKVSFLSALEEYTKKLNTQ.- + Gln->pyro-Glu (N-term Q)
86	140	1127.5565	6759.2953	6759.2788	2.44	1	20	0.013	R.EQLGPVTQEFWDNLEKETEGLRQEMSKDLEEVKAKVQPYLDDFQKKWQEEMELYR.Q
86	140	1130.2218	6775.2871	6775.2737	1.98	1	19	0.02	R.EQLGPVTQEFWDNLEKETEGLRQEMSKDLEEVKAKVQPYLDDFQKKWQEEMELYR.Q + Oxidation (M)
86	140	1130.2220	6775.2883	6775.2737	2.16	1	18	0.024	R.EQLGPVTQEFWDNLEKETEGLRQEMSKDLEEVKAKVQPYLDDFQKKWQEEMELYR.Q + Oxidation (M)
86	140	1356.0651	6775.2891	6775.2737	2.27	1	18	0.023	R.EQLGPVTQEFWDNLEKETEGLRQEMSKDLEEVKAKVQPYLDDFQKKWQEEMELYR.Q + Oxidation (M)
86	140	971.1896	6791.2760	6791.2687	1.08	1	18	0.022	R.EQLGPVTQEFWDNLEKETEGLRQEMSKDLEEVKAKVQPYLDDFQKKWQEEMELYR.Q + 2 Oxidation (M)
86	140	849.9198	6791.3002	6791.2687	4.64	1	18	0.023	R.EQLGPVTQEFWDNLEKETEGLRQEMSKDLEEVKAKVQPYLDDFQKKWQEEMELYR.Q + 2 Oxidation (M)
86	140	752.0398	6759.2928	6759.2788	2.07	1	16	0.03	R.EQLGPVTQEFWDNLEKETEGLRQEMSKDLEEVKAKVQPYLDDFQKKWQEEMELYR.Q
86	140	849.9175	6791.2820	6791.2687	1.97	1	15	0.036	R.EQLGPVTQEFWDNLEKETEGLRQEMSKDLEEVKAKVQPYLDDFQKKWQEEMELYR.Q + 2 Oxidation (M)
240	267	1055.9110	3164.7112	3164.7009	3.25	0	15	0.033	R.QGLLPVLESFKVSFLSALEEYTKKLNTQ.- + Gln->pyro-Glu (N-term Q)
240	267	1061.5855	3181.7347	3181.7274	2.28	0	15	0.037	R.QGLLPVLESFKVSFLSALEEYTKKLNTQ.-
86	140	752.0392	6759.2877	6759.2788	1.31	1	14	0.06	R.EQLGPVTQEFWDNLEKETEGLRQEMSKDLEEVKAKVQPYLDDFQKKWQEEMELYR.Q
86	140	752.0395	6759.2904	6759.2788	1.71	1	14	0.055	R.EQLGPVTQEFWDNLEKETEGLRQEMSKDLEEVKAKVQPYLDDFQKKWQEEMELYR.Q
86	140	1130.2236	6775.2979	6775.2737	3.57	1	14	0.067	R.EQLGPVTQEFWDNLEKETEGLRQEMSKDLEEVKAKVQPYLDDFQKKWQEEMELYR.Q + Oxidation (M)

Score > 14 indicates identity; MC: missed cleavages

Figure S4. ApoA-I epitope identification using digestion at arginine (R) residues.

A. Full chromatogram of each immunoreactive fraction by nano liquid chromatography system.

B. MS/MS spectra of peptides from the fraction 74: QGLLPVLESFKVSFLSALEEYTKKLNTQ; m/z 796.94 (4+); RT: 79.42 min.

C. MS/MS spectra of peptides from the fraction 74: Pyro-QGLLPVLESFKVSFLSALEEYTKKLNTQ; m/z 1056.24 (3+); RT: 79.33 min.

D. Representative mass spectra of peptides (at RT:61.70) present in the immunoreactive fraction 80 by mass spectrometry (LTQ/Orbitrap).

E. MS/MS spectra of peptides from the fraction 80: QGLLPVLESFKVSFLSALEEYTKKLNTQ; m/z 796.69 (4+); RT: 61.76 min.

F. Representative mass spectra of peptides (at RT:70.65) present in the immunoreactive fraction 80 by mass spectrometry (LTQ/Orbitrap).

G. MS/MS spectra of peptides from the fraction 80: Pyro-QGLLPVLESFKVSFLSALEEYTKKLNTQ; m/z 1056.24 (3+); RT:70.57 min.

H-K. Extracted ion chromatograms, using the first C13 isotope, for the peptides: aa240-267: Pyro-QGLLPVLESFKVSFLSALEEYTKKLNTQ ($[M+4H]^{4+}$ 792.44 and $[M+3H]^{3+}$ 1056.24) and aa240-267: QGLLPVLESFKVSFLSALEEYTKKLNTQ ($[M+4H]^{4+}$ 796.94 and $[M+3H]^{3+}$ 1061.91).

L. ApoAI peptides identified in the fraction 80 using the Mascot Distiller tool.

Figure S5. Immunoreactivity compared to the extract ion chromatogram intensity in peptide fractions derived from ApoA-I digested at arginine residues.

The HPLC-fractions with higher presence of these peptides are 74 and 80, as observed in the maximum extracted ion chromatogram (first C13 isotope). In the fraction 80, the two variants eluted in different retention times, the natural variant eluted at RT=61.70min (also in Figure S4D and E), while the pyroglutamic variant eluted at RT=70.65min (also in Figure S4F and G), the same did not happen for the other HPLC fractions, both peptides eluted at the same retention time (Figure 5F and S4B and C). Other peptides derived from miscleavages of ApoA-I were also identified in the fraction 80 (Figure S4L in Supporting information). Therefore, Figure S5 indicates that the level of maximum extracted ion chromatogram intensity for the peptide from the C-terminal helix, independently of the charge state, corresponds to the level of immunoreactivity observed in each one of the HPLC-fractions using samples from patients containing high-titer of autoantibodies against ApoA-I.

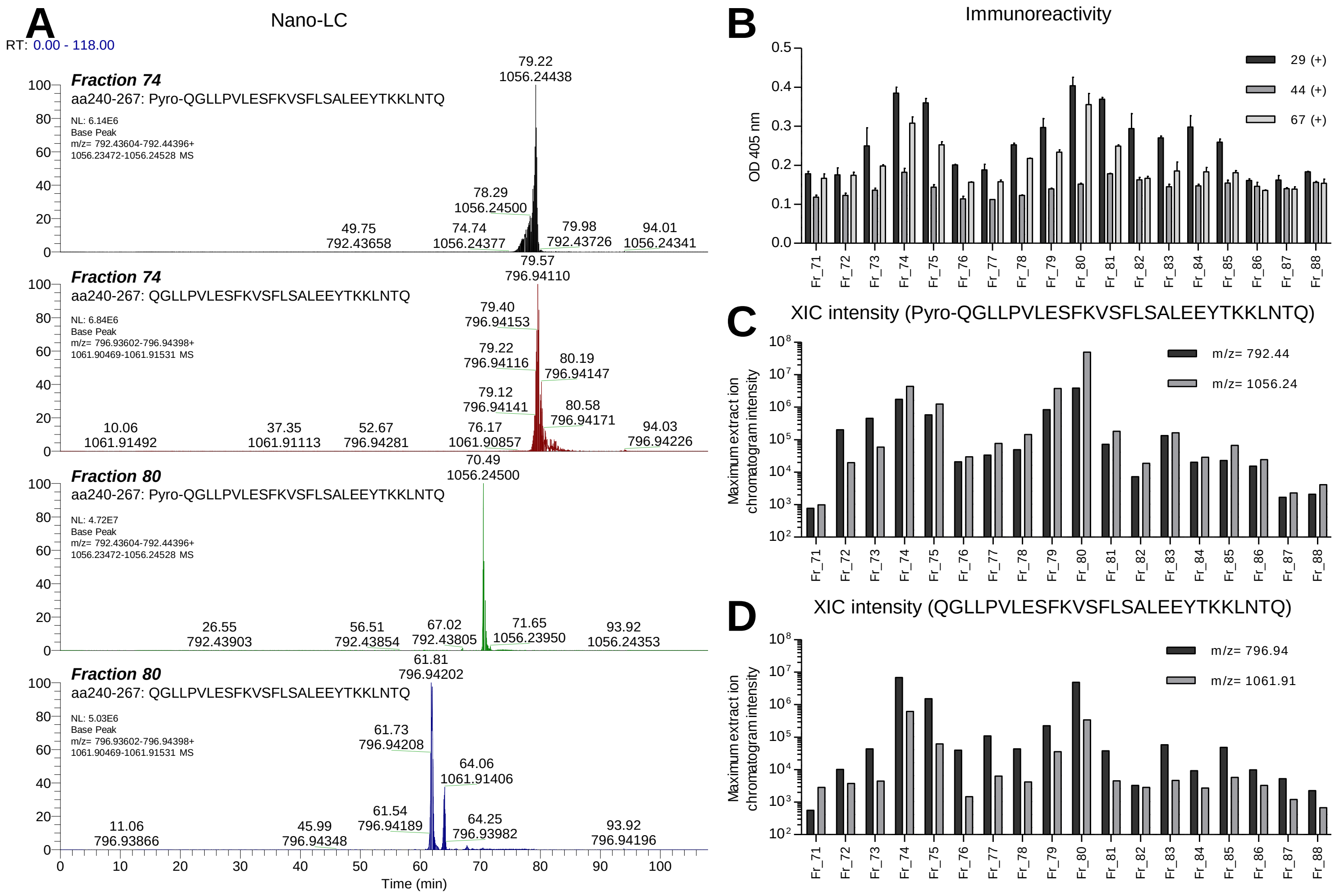

Figure S5. Immunoreactivity compared to the extract ion chromatogram intensity in peptide fractions derived from ApoA-I digested at arginine residues. *(A)* Extracted ion chromatogram of the fraction 74 and 80, using the first C13 isotope, for both peptide C and its pyro-Glu variant. *(B)* Comparison between intensity of the immunoreactivity (OD at 405 nm, arbitrary unit) and maximum extracted ion chromatogram for the peptide C and its pyro-Glu variant in each HPLC fraction.
